# Regulator of calcineurin 1 gene isoform 4 in pancreatic ductal adenocarcinoma regulates the progression of tumor cells

**DOI:** 10.1038/s41388-021-01763-z

**Published:** 2021-04-06

**Authors:** Mengyi Lao, Xiaozhen Zhang, Tao Ma, Jian Xu, Hanshen Yang, Yi Duan, Honggang Ying, Xiaoyu Zhang, Chengxiang Guo, Junyu Qiu, Xueli Bai, Tingbo Liang

**Affiliations:** 1 Department of Hepatobiliary and Pancreatic Surgery, The First Affiliated Hospital, Zhejiang University School of Medicine, Hangzhou, China; 2 Zhejiang Provincial Key Laboratory of Pancreatic Disease, Hangzhou, China; 3 Zhejiang Provincial Innovation Center for the Study of Pancreatic Diseases, Hangzhou, China

**Keywords:** Cancer genetics, Cell growth

## Abstract

Therapeutic strategies to treat pancreatic ductal adenocarcinoma (PDAC) remain unsatisfying and limited. Therefore, it is imperative to fully determine the mechanisms underlying PDAC progression. In the present study, we report a novel role of regulator of calcineurin 1, isoform 4 (RCAN1.4) in regulating PDAC progression. We demonstrated that RCAN1.4 expression was decreased significantly in PDAC tissues compared with that in para-cancerous tissues, and correlated with poor prognosis of patients with pancreatic cancer. In vitro, stable high expression of RCAN1.4 could suppress the metastasis and proliferation and angiogenesis of pancreatic tumor cells. In addition, interferon alpha inducible protein 27 (IFI27) was identified as having a functional role in RCAN1.4-mediated PDAC migration and invasion, while VEGFA play a vital role in RCAN1.4-mediated PDAC angiogenesis. Analysis of mice with subcutaneously/orthotopic implanted xenograft tumors and liver metastasis model confirmed that RCAN1.4 could modulate the growth, metastasis, and angiogenesis of tumors via IFI27/VEGFA in vivo. In conclusion, our results suggested that RCAN1.4 suppresses the growth, metastasis, and angiogenesis of PDAC, functioning partly via IFI27 and VEGFA. Importantly, our results provided possible diagnostic criteria and therapeutic targets for PDAC.

## Introduction

Pancreatic ductal adenocarcinoma (PDAC) is a serious malignancy whose prognosis is very poor. PDAC is the fourth-leading cause of cancer-related death [1]. The overall 5-year survival rate is as low as 6–8% [1, 2]. The major reasons for PDAC’s lethality are late diagnosis, with most patients presenting with locally advanced or metastatic disease at diagnosis, and resistance to chemotherapy. Systemic treatment of PDAC, including surgery and chemotherapy, are still available, such as two combination regimens, FOLFIRINOX (a combination of oxaliplatin, folinic acid, irinotecan, and fluorouracil) and albumin-bound paclitaxel in combination with gemcitabine; however, their overall efficacy remains poor [3–5]. Therefore, there is an urgent need to identify more causative genes and their associated molecular pathways responsible for the progression and metastasis of PDAC.

Down syndrome is the most common genetic disorder in humans, which results from an extra copy of part or all of chromosome 21. Epidemiological studies indicate that individuals with Down syndrome experience decreased incidence and mortality for many types of solid tumors [6–9]. For example, the Down syndrome population display a sevenfold reduced incidence of pancreatic cancer compared with that in the general population [6]. The *RCAN1* (encoding regulator of calcineurin 1, also known as Down syndrome critical region 1) gene is located on chromosome 21 and encodes an endogenous inhibitor of calcineurin [10, 11]. Several studies have demonstrated that overexpression of *RCAN1* in vascular endothelium cells contributes to a tumor protective effect by attenuating tumor angiogenesis mediated by vascular endothelial growth factor (VEGF) via inhibition of the calcineurin pathway [10–13].

The roles of RCAN1 in suppressing tumor growth and blocking metastasis have been identified in many types of cancer. However, its function in PDAC development is completely unknown. Lee et al. demonstrated that in Pdx-1-Cre;LSL-Kras^G12D^ mice, a genetically engineered mouse model of human PDAC, *RCAN1* trisomy could suppress the progression of early pancreatic intraepithelial neoplasia lesions by attenuating the calcineurin-nuclear localization of nuclear factor of activated T cells (NFAT) axis, together with inhibition of cell proliferation in the neoplastic ductal epithelium [14]. In addition, there are two main isoforms expressed from *RCAN1*, RCAN1.1 and RCAN1.4. The expression of RCAN1.1 is constitutive, whereas the expression of RCAN1.4 is induced by certain physiological changes. RCAN1.4 competitively inhibits the phosphatase calcineurin and is involved in the regulation of calcineurin/NFAT signaling [15, 16]. RCAN1.4 functions as a suppressor of cancer progression by inhibiting the activity of NFAT in hepatocellular carcinoma (HCC) [17], thyroid cancer [18], and renal cell carcinoma (RCC) [19]. However, RCAN1.4’s role in PDAC progression is unknown. The present study aimed to investigate the contributions and detailed mechanism of RCAN1.4 toward PDAC growth and metastasis, angiogenesis, and to provide insights into the clinical prognosis and potential therapeutic targets in PDAC.

## Results

### RCAN1.4 is a candidate tumor suppressor that is associated with poor prognosis in patients with pancreatic cancer

**Fig. 1:**
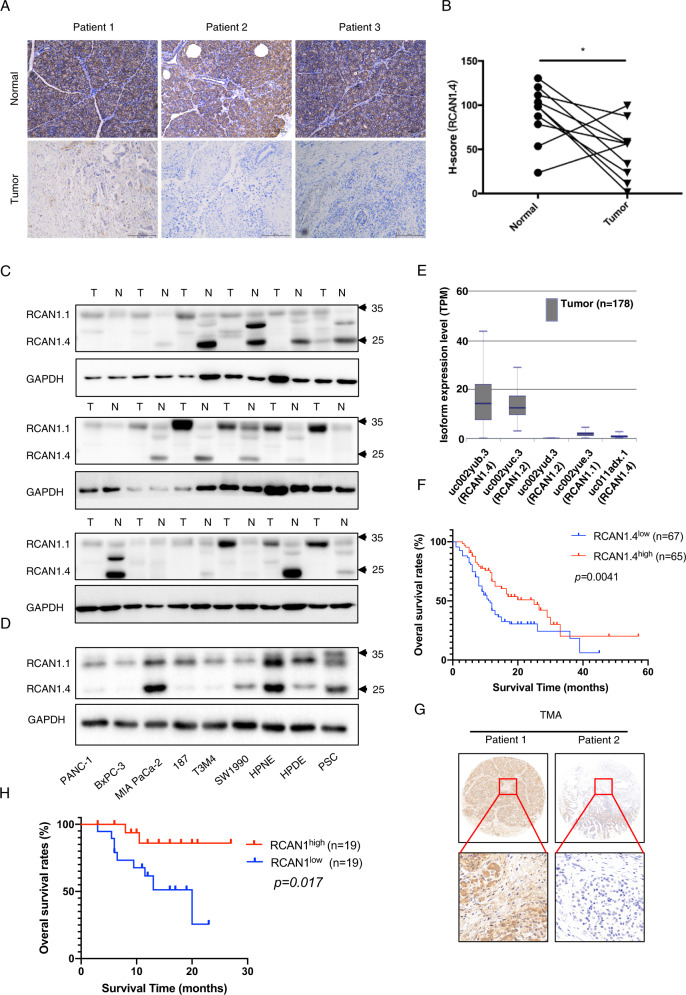
RCAN1.4 downregulation and its significance in the prognosis of pancreatic cancer. **A** The representative images of paired tumorous (T) and normal (N) pancreatic tissues were individually subjected to immunohistochemical (IHC) staining to detect RCAN1.4 levels. **B** The H-score of the expression of RCAN1.4 between paired tumorous and normal pancreatic tissues (*n* = 10). **C** Immunoblotting analysis was also performed between paired tumorous and normal pancreatic tissues (*n* = 18). **D** Immunoblotting analysis of RCAN1.4 protein levels among human pancreatic cancer cell lines (PANC-1, BxPC-3, MIA PaCa-2, 187, T3M4, and SW1990), normal pancreatic ductal cells (HPNE and HPDE), and pancreatic cancer-associated fibroblasts (PSCs). **E** TCGA analysis showed the expression levels of RCAN1 transcript isoforms in PAAD tissues using the ISOexpresso website (http://wiki.tgilab.org/ISOexpresso/). **F** RCAN1.4 levels and their association with OS was determined using Kaplan–Meier analysis. Samples from 103 patients with PAAD were subjected to RCAN1.4 IHC staining. RCAN1.4 levels and their association with OS was determined using Kaplan–Meier analysis in a PDAC tissue microarray. **G** Representative image of the IHC staining of RCAN1.4 in the PDAC tissue microarray (TMA). **H** Serum RCAN1 level and their association with OS determined using Kaplan–Meier analysis. Serum RCAN1 from 38 patients with PAAD were detected with ELISA.

The expression of RCAN1.4 between cancerous and para-cancerous tissues from patients with PDAC was compared using IHC. We found that RCAN1.4 levels were reduced significantly in PDAC tissues compared with those in para-cancerous tissues (Fig. 1A, B). The expression of RCAN1.4, not RCAN1.1, was significantly higher in the matched para-cancerous tissues than that in cancerous tissues using western blotting (Fig. 1C). In addition, to determine the endogenous levels of RCAN1.4 in pancreatic cancer cells, a set of confirmed pancreatic cancer cell lines was screened using western blotting. As expected, RCAN1.4 showed low expression in most PDAC cell lines, except MIA PaCa-2, while high expression was observed in normal pancreatic cell lines. The RCAN1.1 in all cell line was similar (Fig. 1D). RCAN1 can be expressed as 3 mRNA isoforms (RCAN1.1 (uc002yue.3), RCAN1.2 (uc002yuc.3, uc002yud.3), and RCAN1.4 (uc002yub.3, uc011adx.1). TCGA analysis showed that RCAN1.4 was the major RCAN1 isoform in the PDAC tissues based on the median TPM value, and RCAN1.1 and RCAN1.2, were found at low levels (Fig. 1E). Kaplan–Meier survival analysis showed that patients with PDAC with low RCAN1.4 protein levels had worse overall survival (OS) than those with high RCAN1.4 protein levels, as analyzed using the tumor microarray (Fig. 1F, G). A clinical association study revealed that low RCAN1.4 expression was only associated significantly with vascular invasion (*p* = 0.028), not tumor–node–metastasis (TNM) stage and grade (Table 1). Similarity, TCGA analysis showed the mRNA levels of RCAN1 were no significantly associated with grade and TNM stage (Supplementary Fig. 1A, B). The Cox regression analysis was performed to confirmed that the RCAN1.4 protein level can serve as an independent prognostic indicator of PDAC (Table 2 and Supplementary Fig. 1C). The Kaplan–Meier survival analysis further revealed that patients with low serum RCAN1 level had a shortened OS compared to those with high level (Fig. 1H). Statistical analysis revealed that low serum RCAN1 level was associated significantly with vascular invasion (*p* = 0.011) and a higher serum CA19-9 level (*p* = 0.028) (Table 3). Taken together, our results indicated that RCAN1.4 is a tumor suppressor that is associated with poor prognosis in patients with pancreatic cancer.

**Table 1:** Correlation between the protein levels of RCAN1.4 with clinicopathological features in 153 PDAC patients.

Variable	TMA RCAN1.4		*p* value
	Low (*H*-score ≤ 120, *n* = 76)	High (*H*-score > 120, *n* = 77)	
Gender			0.674
Male, *n* (%)	45 (59.21)	43 (55.84)	
Female, *n* (%)	31 (40.79)	34 (44.16)	
Age, years			0.501
>60, *n* (%)	67 (88.16)	65 (84.42)	
≤60, *n* (%)	9 (11.84)	12 (15.58)	
BMI, kg/m^2^ (IQR)	23.15 (20.31–24.49)	22.04 (20.55–23.62)	0.177
Tumor size			0.435
<3 mm, *n* (%)	24 (31.58)	28 (36.36)	
≥3 mm, *n* (%)	43 (56.58)	38 (49.35)	
Missing, *n* (%)	9 (11.84)	11 (14.29)	
TNM stage			0.071
I, *n* (%)	23 (30.26)	13 (16.88)	
II, *n* (%)	27 (35.53)	40 (51.95)	
III–IV, *n* (%)	24 (31.58)	23 (29.87)	
Missing, *n* (%)	2 (2.63)	1 (1.30)	
Vascular invasion			**0.028**
Yes, *n* (%)	42 (55.26)	29 (37.66)	
No, *n* (%)	33 (43.42)	47 (61.04)	
Missing, *n* (%)	1 (1.32)	1 (1.30)	
Nerve invasion			0.764
Yes, *n* (%)	53 (69.74)	52 (67.53)	
No, *n* (%)	22 (28.95)	24 (31.17)	
Missing, *n* (%)	1 (1.32)	1 (1.30)	
Serum CA125, U/ml			0.774
≥35, *n* (%)	17 (22.37)	19 (24.68)	
<35, *n* (%)	51 (67.11)	51 (66.23)	
Missing, *n* (%)	8 (10.53)	7 (9.09)	
Serum CA19-9, U/ml			0.969
≥37, *n* (%)	61 (80.26)	62 (80.52)	
<37, *n* (%)	14 (18.42)	14 (18.18)	
Missing, *n* (%)	1 (1.32)	1 (1.30)	
Serum CEA, U/ml			0.555
≥5, *n* (%)	22 (28.95)	26 (33.77)	
<5, *n* (%)	47 (61.84)	45 (58.44)	
Missing, *n* (%)	7 (9.21)	6 (7.79)	
Tumor differentiation			0.586
Well, *n* (%)	3 (3.95)	5 (6.49)	
Moderate, *n* (%)	45 (59.21)	47 (61.04)	
Poor, *n* (%)	26 (34.21)	21 (27.27)	
Missing, *n* (%)	2 (2.63)	4 (5.19)	
Recurrence			0.982
Yes, *n* (%)	39 (51.32)	42 (54.55)	
No, *n* (%)	15 (19.74)	16 (20.78)	
Missing, *n* (%)	22 (28.95)	21 (27.27)	

Bold value indicates statistical significance.

**Table 2:** Multivariate analyses of factors associated with survival in 132 PDAC patients.

	Multivariate	
Variable	HR (95% CI)	*p* value
Vascular invasion	1.937 (1.216–3.086)	**0.005**
TNM stage	1.426 (1.037–1.963)	**0.029**
RCAN1.4: low vs. high	2.173 (1.360–3.470)	**0.001**

Bold values indicate statistical significance.

**Table 3:** Correlation between the serum levels of RCAN1 with clinicopathological features in 50 PDAC patients.

Variable	Serum RCAN1.4		*p* value
	Low (≤4.41 ng/ml, *n* = 25)	High (>4.41 ng/ml, *n* = 25)	
Gender			0.087
Male, *n* (%)	11 (44.00)	17 (68.00)	
Female, *n* (%)	14 (56.00)	8 (32.00)	
Age, years			0.157
>60, *n* (%)	22 (88.00)	18 (72.00)	
≤60, *n* (%)	3 (12.00)	7 (28.00)	
BMI, kg/m^2^ (IQR)	22.61 (20.05-25.51)	21.67 (19.84-24.33)	0.66
Tumor size			0.208
<3 mm, *n* (%)	5 (20.00)	9 (36.00)	
≥3 mm, *n* (%)	20 (80.00)	16 (64.00)	
Missing, *n* (%)	0	0	
TNM stage			0.216
I, *n* (%)	3 (12.00)	8 (32.00)	
II, *n* (%)	17 (68.00)	14 (56.00)	
III–IV, *n* (%)	5 (20.00)	3 (12.00)	
Missing, *n* (%)	0	0	
Vascular invasion			**0.011**
Yes, *n* (%)	17 (68.00)	8 (32.00)	
No, *n* (%)	8 (32.00)	17 (68.00)	
Missing, *n* (%)	0	0	
Nerve invasion			0.185
Yes, *n* (%)	21 (84.00)	17 (68.00)	
No, *n* (%)	4 (16.00)	8 (32.00)	
Missing, *n* (%)	0	0	
Serum CA125, U/ml			0.291
≥35, *n* (%)	5 (20.00)	8 (32.00)	
<35, *n* (%)	20 (80.00)	16 (64.00)	
Missing, *n* (%)	0	0	
Serum CA19-9, U/ml			**0.021**
≥37, *n* (%)	24 (96.00)	18 (72.00)	
<37, *n* (%)	1 (4.00)	7 (28.00)	
Missing, *n* (%)	0	0	
Serum CEA, U/ml			0.93
≥5, *n* (%)	9 (36.00)	12 (48.00)	
<5, *n* (%)	16 (64.00)	13 (52.00)	
Missing, *n* (%)	0	0	
Tumor differentiation			0.203
Well, *n* (%)	0	3 (12.00)	
Moderate, *n* (%)	16 (64.00)	14 (56.00)	
Poor, *n* (%)	9 (36.00)	8 (32.00)	
Missing, *n* (%)	0	0	
Recurrence			0.083
Yes, *n* (%)	18 (72.00)	12 (48.00)	
No, *n* (%)	7 (28.00)	13 (52.00)	
Missing, *n* (%)	0	0	

Bold values indicate statistical significance.

### RCAN1.4 plays a tumor suppressive role in PDAC

**Fig. 2:**
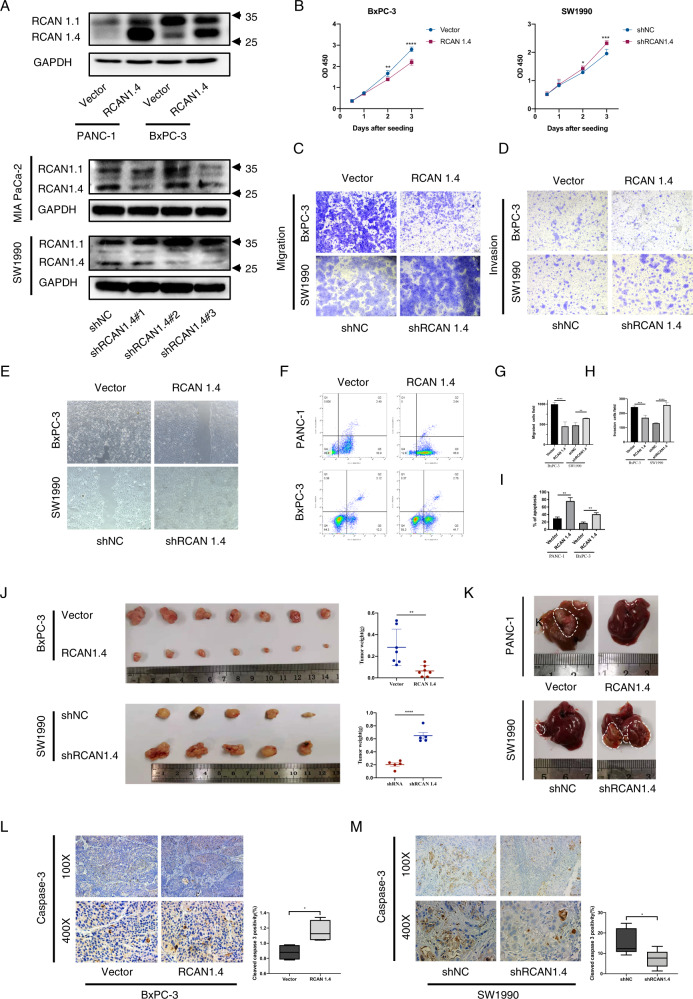
RCAN1.4 shows tumor suppressor functions in PDAC. **A** Immunoblot in BxPC-3 and PANC-1 cells stably overexpressing RCAN1.4 (top), and in SW1990 and MIA PaCa-2 cells knockdown RCAN1.4 (bottom). **B** Effects of RCAN1.4 overexpression and knockdown on proliferation of BxPC-3 (left) and SW1990 (right) cells by CCK-8 assay. **C**, **G** Effects of RCAN1.4 overexpression and knockdown on migration of BxPC-3 and SW1990 cells. Representative images (**C**) and quantification (**G**) of migration of RCAN1.4 overexpression BxPC-3 and RCAN1.4 knockdown SW1990 cells. Scale bar = 100 μm. Effects of RCAN1.4 overexpression and knockdown on invasion of BxPC-3 and SW1990 cells. Representative images (**D**) and quantification (**H**) of invasion of RCAN1.4 overexpression BxPC-3 and RCAN1.4 knockdown SW1990 cells. Scale bar = 100 μm. **E** Representative images of wound healing assay of BxPC-3 and SW1990 cells to detect cell migration. Representative images (**F**) and quantification (**I**) of apoptosis was performed in RCAN1.4 overexpression PANC-1 and BxPC-3 cells. **J** PDAC cells were orthotopic transplanted into the pancreas of nude mice. Tumor representative images (left) and tumor weight (right) of RCAN1.4 overexpression BxPC-3 (top) and RCAN1.4 knockdown SW1990 (bottom) cells in immunodeficient mice. **K** PDAC cells were injected into the spleen of nude mice. Hepatic metastatic tumor representative images of RCAN1.4 overexpression PANC-1 (top) and RCAN1.4 knockdown SW1990 (bottom) cells. **L**, **M** Representative images (left) and quantification (right) of cleaved caspase-3 staining for the tumors. Results are presented as the mean ± SD from one representative experiment. Error bars, ±SD (determined using a two-tailed *t*-test, ns no significance, *p < 0.05, **p < 0.01, ***p < 0.001, ****p < 0.0001).

As show in Fig. 1D, MIA PaCa-2 and SW1990 cells expressed higher endogenous levels of RCAN1.4 than the other cell lines (PANC-1, BxPC-3, 187, and T3M4). Therefore, MIA PaCa-2 and SW1990 cells were selected to create stable *RCAN1.4* knockdown cells using short hairpin RNAs specifically targeting *RCAN1.4* (shRCAN1.4). RCAN1.4 was overexpressed in PANC-1 and BxPC-3 cells from a *RCAN1.4* overexpression lentivirus. Efficiency of overexpression and knockdown was detected using western blotting (Fig. 2A).

Overexpression of RCAN1.4 significantly inhibited the proliferation of BxPC-3 and PANC-1 cells, whereas knockdown of RCAN1.4 significantly promoted the proliferation of SW1990 and MIA PaCa-2 cells in vitro using CCK-8 assay (Fig. 2B and Supplementary Fig. 2A). Similarly, EdU assays was used to determine the effect of RCAN1.4 on cell proliferation (Supplementary Fig. 2B). The effects of RCAN1.4 on migration and invasion were evaluated using Transwell assays and wound healing assay. Overexpression of RCAN1.4 significantly inhibited the migration and invasion of BxPC-3 and PANC-1 cells, whereas knockdown of RCAN1.4 significantly promoted the migration and invasion of SW1990 and MIA PaCa-2 cells in vitro (Fig. 2C, D, G, H and Supplementary Fig. 2C, D). This result was confirmed in SW1990, and BxPC-3 cells using the wound healing assay (Fig. 2E). Next, the effect of RCAN1.4 overexpression on tumor apoptosis was investigated using flow cytometry. The results revealed that overexpression of RCAN1.4 significantly promoted tumor cell apoptosis (Fig. 2F, I). In conclusion, the above results revealed that RCAN1.4 could regulate tumor cell proliferation, metastasis, and apoptosis in vitro.

To determine whether RCAN1.4 could regulate tumor cell growth and metastasis in vivo, we established an orthotopic injection model by injection of PDAC cells into pancreas of male 6–8-week-old nude mice. Overexpression of RCAN1.4 significantly reduced the size of pancreatic tumors in the orthotopic pancreas compared with the control group in vivo, whereas RCAN1.4 knockdown significantly increased the tumor size (Fig. 2J). As well, the consistent results were obtained in the subcutaneous injection model (Supplementary Fig. 3A–C). The liver metastasis model was established by injection of PDAC cells into spleen of male 6–8-week-old nude mice. Overexpression of RCAN1.4 significantly reduced the number of metastatic liver nodules. And knockdown of RCAN1.4 significantly increased the number of metastatic liver nodules (Fig. 2K). Cleaved caspase-3 was stained to assess tumor apoptosis, and the IHC results showed that in RCAN1.4 overexpression BxPC-3 tumors have significantly more cleaved caspase-3-positive cells compared with the relative control group. In contrast, RCAN1.4 knockdown SW1990 tumors had fewer cleaved caspase-3-positive cells (Fig. 2L, M). Next, in vivo cell proliferation in tumor sections was assessed using Ki-67 staining. However, no difference between the control and RCAN1.4 overexpression or control and RCAN1.4 knockdown xenografts was observed (Supplementary Fig. 4A, B).

### RCAN1.4 blocks calcineurin–NFAT1 signaling pathway in PDAC

Several studies have demonstrated that RCAN1.4 is an endogenous inhibitor of Calcineurin–NFAT1 Signaling in a wide variety of tumor. The tumor suppressive function of RCAN1.4 is performed by specifically blocking calcineurin-mediated nuclear activated factor of activated T cells (NFAT) localization and transcriptional activity [17]. NFAT genes are also involved in the development and metastasis of PDAC.

**Fig. 3:**
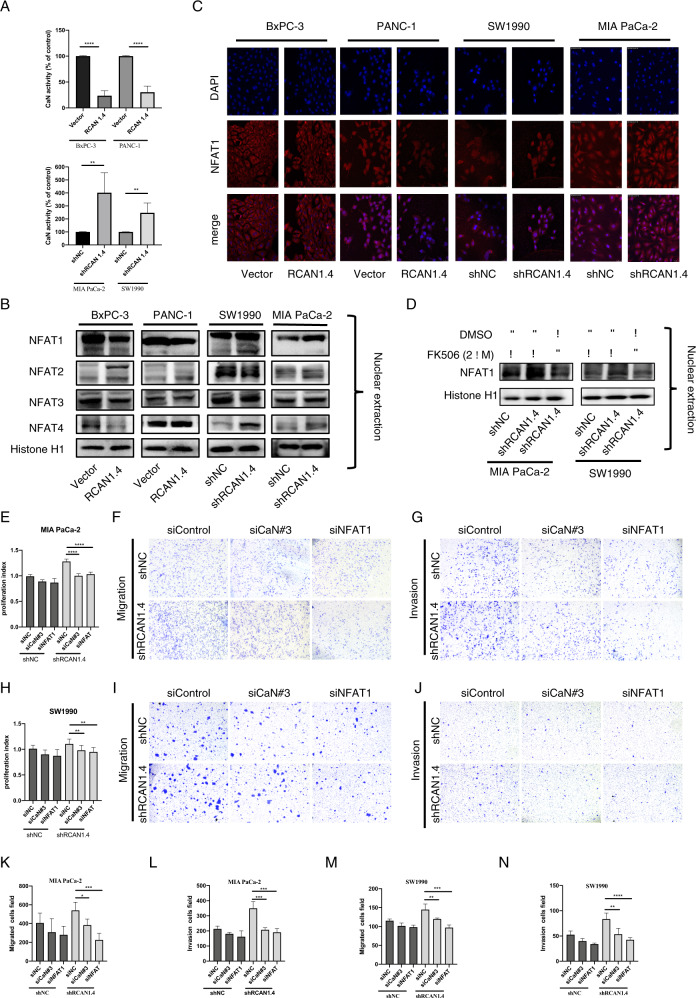
RCAN1.4 blocked calcineurin-mediated NFAT1 signaling pathway in PDAC. **A** The activity of calcineurin in PDAC cells was detected by calcineurin assays. The control cells were set as 100%. **B** Immunoblotting analysis of Nuclear levels of NFAT subtypes (NFAT1–4) were detected. **C** PDAC cells were stained with fluorescent antibodies against NFAT1 (red) or with DAPI (blue). Representative confocal immunofluorescence images are shown. Scale bar 25 μm. **D** The regulatory role of RCAN1.4 in calcineurin–NFAT1 signaling was confirmed by treatment with calcineurin inhibitor (FK506 2 μM) by western blotting. **E**–**N** RCAN1.4 knockdown SW1990 cells or RCAN1.4 knockdown MIA PaCa-2 cells were transfected with calcineurin (CaN#3) siRNAs, or NFAT1 siRNAs. Quantification of cell viability of images per group was shown by CCK-8 assay (**E**, **H**). Representative images (**F**, **I**) and quantification (**K**, **M**) of migratory cells per group was shown. Representative images (**G**, **J**) and quantification (**L**, **N**) of invasion cells per group was shown. Results are presented as the mean ± SD from one representative experiment. Error bars, ±SD (determined using a two-tailed *t*-test, ns no significance, *p < 0.05, **p < 0.01, ***p < 0.001, ****p < 0.0001).

Therefore, the phosphatase activity of calcineurin was measured in PDAC cells after stable overexpression or knockdown of RCAN1.4. As expected, the activity of calcineurin was significantly reduced in RCAN1.4 overexpression BxPC-3 and PANC-1 cells compared with those control groups, while increased in RCAN1.4 knockdown SW1990 and MIA PaCa-2 cells (Fig. 3A). Furthermore, we extracted the nuclear proteins and cytoplasmic proteins in PDAC cells after stable overexpression or knockout of RCAN1.4. Western blotting demonstrated that the nuclear accumulation of NFAT1 was obviously blocked when RCAN1.4 was overexpressed, while promoted when RCAN1.4 was knockdown in PDAC cells by detecting the NFAT1 level in nucleoprotein (Fig. 3B and Supplementary Fig. 5).

The results of immunofluorescence further confirmed RCAN1.4 inhibited NFAT1 nuclear translocation (Fig. 3C). Next, RCAN1.4 knockdown PDAC cells were treated with a calcineurin inhibitor (FK506). The results showed the RCAN1.4-mediated nuclear accumulation of NFAT1 can be reversed in PDAC cells (Fig. 3D).

Next, RCAN1.4 knockdown SW1990 and MIA PaCa-2 cells were transfected with a set of siRNAs to silence calcineurin and NFAT1 expression (Supplementary Fig. 6A, B). The results shown that the promoted effects, which was upregulated by RCAN1.4 knockdown, on proliferation, migration, and invasion can be reversed by silencing calcineurin and NFAT1 expression (Fig. 3E–N).

### *IFI27*, a direct transcriptional target of NFAT1, has a functional role in RCAN1.4-mediated PDAC progression

**Fig. 4:**
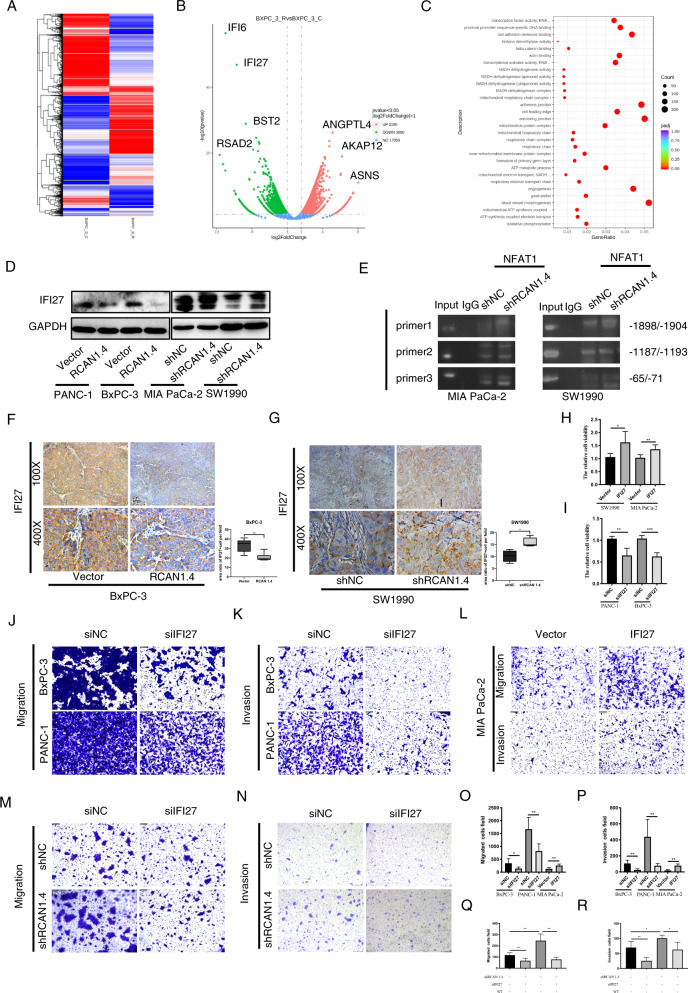
IFI27 is a direct transcriptional target of NFAT1 and essential for RCAN1.4-mediated PDAC progression. **A**, **B** Heatmap and volcano plot visualization of differentially expressed genes between RCAN1.4 overexpression BxPC-3 and WT-BxPC-3 cells from the RNA-seq data. **C** GO enrichment analysis was performed for differentially expressed genes between RCAN1.4 overexpression BxPC-3 and WT-BxPC-3 cells from the RNA-seq data. **D** The protein levels of IFI27 decreased in RCAN1.4 overexpression PANC-1 and BxPC-3 cells and increased in RCAN1.4 knockdown MIA PaCa-2 and SW1990 cells using western blotting. **E** NFAT1 increasingly bound three potential binding sites in the IFI27 promotor by ChIP assay. **F**, **G** The representative images and immunohistochemical staining quantification analysis of IFI27 were performed respectively in RCAN1.4 overexpression BxPC-3 and WT-BxPC-3, RCAN1.4 knockdown SW1990 and WT-SW1990 orthotopic tumors in nude mice. **H**, **I** Quantification of CCK-8 in IFI27-knockdown PANC-1 and BxPC-3 cells, and IFI27 overexpression SW1990 and MIA PaCa-2 cells to detect cell viability. **J**, **K** Representative images of migration and invasion of *IFI27*-knockdown PANC-1 and BxPC-3 cells. Scale bar = 100 μm. **L** Representative images of migration and invasion of *IFI27* overexpression MIA PaCa-2 cells. Scale bar = 100 μm. **M**, **N** Representative images of migration and invasion of *IFI27*-knockdown of RCAN1.4 KD-SW1990 and WT-SW1990 cells. Scale bar = 100 μm. **O**, **P** The quantification of migration and invasion of *IFI27*-knockdown PANC-1 and BxPC-3 cells, and *IFI27* overexpression MIA PaCa-2 cells. **Q**, **R** The quantification of migration and invasion of *IFI27*-knockdown of RCAN1.4 KD-SW1990 and WT-SW1990 cells. Results are presented as mean ± SD from one representative experiment. Error bars, ±SD (determined using a two-tailed *t*-test, ns no significance, *p < 0.05, **p < 0.01, ***p < 0.001, ****p < 0.0001).

To investigate the detailed mechanism of RCAN1.4-mediated PDAC progression, RNA-seq was performed between RCAN1.4 overexpression BxPC-3 and WT-BxPC-3 cells. Heatmaps and volcano plots of the differentially expressed genes are shown in Fig. 4A, B. Gene ontology enrichment analysis was performed between RCAN1.4 overexpression BxPC-3 and WT-BxPC-3 from the RNA-seq data. The results showed that the gene ratio and the number of differentially expressed genes associated with blood vessel morphogenesis, anchoring junction, adherens junctions, transcription factor activity, and transcriptional activator factor increased significant between RCAN1.4-OE and WT-BxPC-3 cells (Fig. 4C). To determine whether any of the differentially expressed genes were regulated by RCAN1.4 in PDAC cell lines, qRT-PCR experiments were performed for validation. The top five upregulated and downregulated genes, and ten dis-regulated genes were validated by qRT-PCR (Supplementary Fig. 7A, B). Furthermore, six of the ten differential genes were validated by western blotting (Supplementary Fig. 7C). Only IFI27 was both downregulated in RCAN1.4 overexpression BxPC-3 and PANC-1 cells, and upregulated in RCAN1.4 knockdown SW1990 and MIA PaCa-2 cells (Fig. 4D). We predicted putative NFAT1-binding sites within the *IFI27* promoter regions with a length of 2000 bp with JASPAR: http://jaspar.genereg.net. Surprisingly, NFAT1 increasingly bound three potential binding sites in the *IFI27* promotor when RCAN1.4 was knockdown in PDAC cells by using chromatin immunoprecipitation (ChIP) assay (Fig. 4E). IHC in the in vivo xenografts showed that IFI27 expression was significantly decreased in RCAN1.4 overexpression BxPC-3 orthotopic tumors compared with that the relative control group, while increased in RCAN1.4 knockdown SW1990 orthotopic tumors (Fig. 4F, G).

Then, the role of endogenous IFI27 in tumor invasion, migration, and growth was investigated in vitro. CCK-8 and Transwell assays revealed that IFI27 overexpression promoted the invasion, migration, and proliferation of PDAC cells (Fig. 4H, L, O, P). Next, to reveal if IFI27 is involved in the mechanism of RCAN1–4’s function in cancer cells, migration and invasion assays were performed in RCAN1.4 knockdown SW1990 cells overexpressing IFI27. As expected, IFI27 could revert the promotion of migration and invasion induced by RCAN1.4 knockdown in PDAC tumor cells (Fig. 4M, N, Q, R). These findings suggested that IFI27 is involved in RCAN1.4-mediated invasion, migration, and growth of PDAC.

**Fig. 5:**
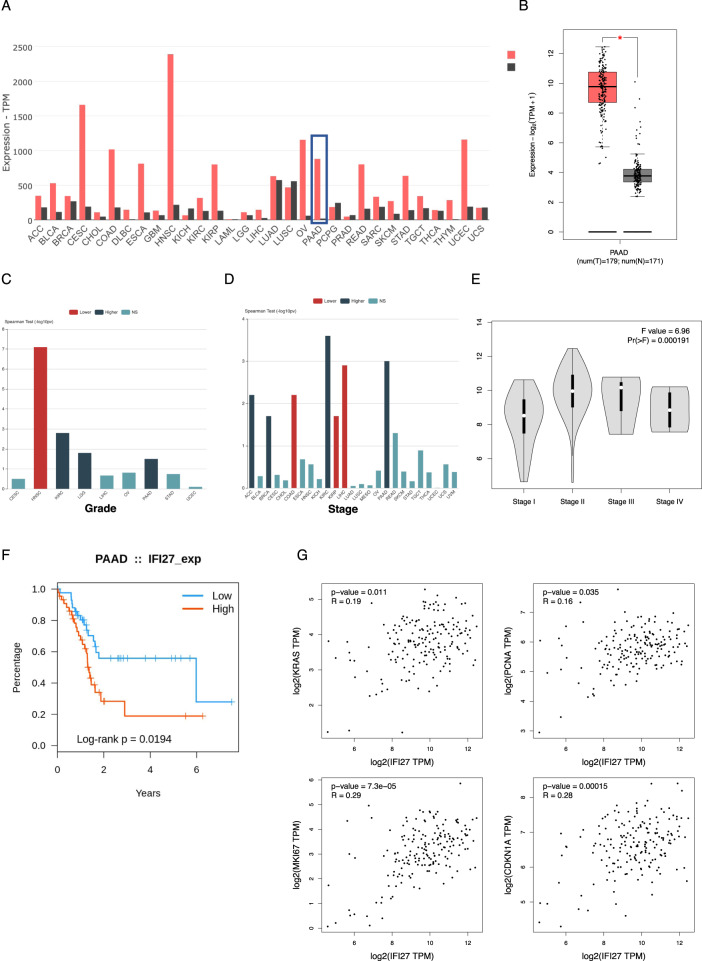
The involvement of IFI27 in PAAD. **A** Summary of the expression landscape of *IFI27* proteins in multiple cancer types. **B** Relative expression levels of *IFI27* in pancreatic cancer analyzed individually using large-scale RNA-Seq datasets of PAAD from the TCGA database (*n* = 350). **C**, **D** Association between the expression of *IFI27*, and tumor stage and grade using large-scale RNA-Seq datasets in multiple cancer types from the TCGA database. **E** Association between the expression of *IFI27* and tumor stage using large-scale RNA-Seq datasets of pancreatic cancer from the TCGA database. **F** Overall survival (OS) of patients with pancreatic cancer with high or low expression of *IFI27* from the TCGA database. **G** Bioinformatic analysis of the correlation between *IFI27* and immune effector molecules using datasets from the TCGA database. TPM transcript per million.

To investigate whether IFI27 is a crucial regulator in PDAC, we detected the IFI27 protein level in a PDAC tissue microarray. There was no significant difference in IFI27 protein levels and OS in patients with PDAC by Kaplan–Meier survival analysis (*p* = 0.053) (Supplementary Fig. 7D). However, we performed bioinformatic analysis for IFI27. The results from TCGA database inferred that *IFI27* expression is high in pancreatic cancer compared with that in normal tissues (Fig. 5A, B). Clinical factors analysis revealed that a high expression of IFI27 was associated significantly with poor OS, stage, and grade of PDAC (Fig. 5C–F). Then in the analysis of the association between *IFI27* and carcinogenesis genes, we found that *IFI27* was strongly and positively associated with *KRAS* (KRAS proto-oncogene, GTPase), *PCNA* (proliferating cell nuclear antigen), *MKI67* (marker of proliferation Ki-67), and *CDKN1A* (cyclin dependent kinase inhibitor 1A) (Fig. 5G).

### *VEGFA*, another direct transcriptional target of NFAT1, involved in RCAN1.4-mediated angiogenesis

**Fig. 6:**
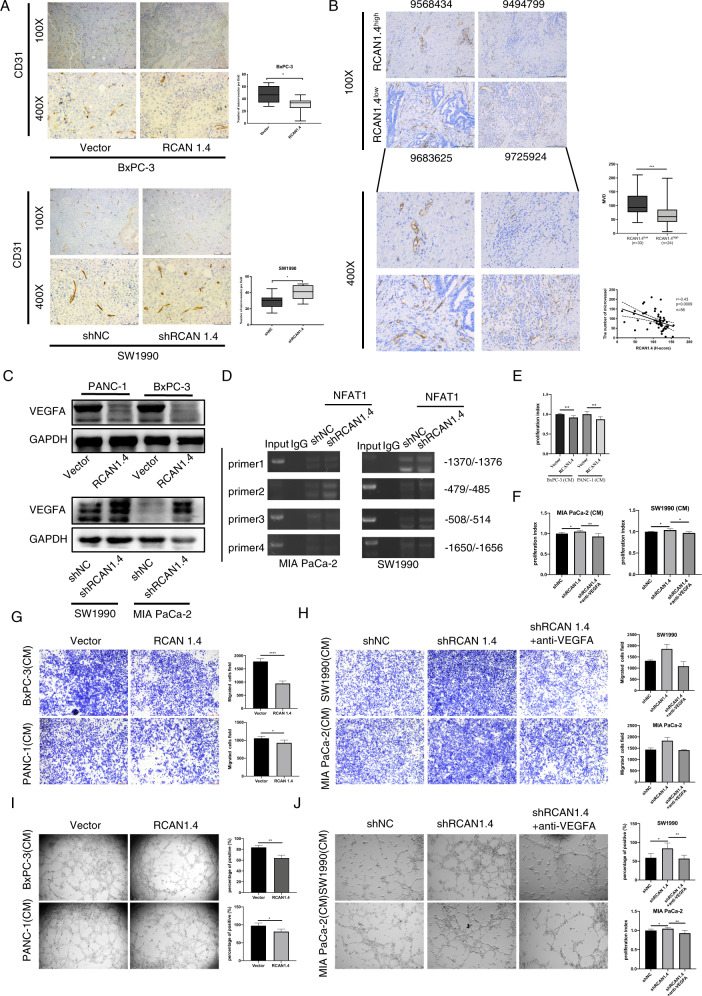
The tumor angiogenesis suppressive effects of RCAN1.4 in PDAC cells by VEGFA pathway. **A** The representative images and immunohistochemical staining quantification analysis of CD31 were performed, respectively, in RCAN1.4 overexpression BxPC-3 and WT-BxPC-3, RCAN1.4 knockdown SW1990, and WT-SW1990 orthotopic tumors in nude mice. Scale bar = 100 μm. **B** The association between RCAN1.4 expression and MVD in human PDAC tissues. Representative images and quantifications of RCAN1.4-high expression and RCAN1.4-low expression pancreatic tissues were individually subjected to CD31 staining. **C** The protein levels of VEGFA decreased in RCAN1.4 overexpression PANC-1 and BxPC-3 cells and increased in RCAN1.4 knockdown MIA PaCa-2 and SW1990 cells using western blotting. **D** NFAT1 increasingly bound four potential binding sites in the IFI27 promotor site by ChIP assay. **E**, **F** Proliferation of HUVECs were analyzed after incubating with CM or CM with VEGFA neutralizing antibodies by CCK-8 assay. **G**, **H** Representative images and quantification of migration of HUVCE after incubating with CM or CM with VEGFA neutralizing antibodies. Scale bar = 100 μM. **I**, **J** Representative images and quantification of tube formation induced after incubating with CM or CM with VEGFA neutralizing antibodies. Scale bar = 100 μM. Results are presented as mean ± SD from one representative experiment. Error bars, ±SD (determined using a two-tailed *t*-test, ns no significance, *p < 0.05, **p < 0.01, ***p < 0.001, ****p < 0.0001).

VEGFA plays a central role in tumor angiogenesis. Jin et al. has confirmed that RCAN1.4 negatively regulated VEGFA expression was associated with angiogenesis in HCC [17]. The analysis of RNA-seq showed *VEGFA* were altered after RCAN1.4 overexpression in BxPC-3 cell (log2 fold change = −2.48). We measured the tumor angiogenesis in orthotopic tumor sections using CD31 staining. The results indicated that RCAN1.4 overexpression tumors had less microvessels compared with the relative control group, while RCAN1.4 knockdown tumors had more microvessels (Fig. 6A). Furthermore, we analyzed the associations between RCAN1.4 protein levels and MVD in tumor tissues from 56 patients with PDAC. Interestingly, there was negative correlation between the protein level of RCAN1.4 and MVD (Fig. 6B). Western blotting demonstrated that VEGFA was downregulated in RCAN1.4 overexpression BxPC-3 and PANC-1 cells, and upregulated in RCAN1.4 knockdown SW1990 and MIA PaCa-2 cells (Fig. 6C). Meanwhile, we predicted putative NFAT1-binding sites within the *VEGFA* promoter regions with a length of 2000 bp with JASPAR: http://jaspar.genereg.net. NFAT1 increasingly bound four potential binding sites in the VEGFA promotor when RCAN1.4 was knockdown in PDAC cells by using ChIP assay (Fig. 6D).

To determine the effect of RCAN1.4 on angiogenesis in vitro, the human umbilical vein endothelial cells (HUVECs) was cultivated with PDAC cells. The proliferation and migration of HUVECs were inhibited when treated with conditioned medium (CM) from PDAC cells that stably overexpressed RCAN1.4 compared with the control CM (Fig. 6E, G). On the contrary, the proliferation and migration of HUVECs were promoted when treated with CM from RCAN1.4 knockdown PDAC cells. These promoted effects can be reversed by treating VEGFA neutralizing antibody (Fig. 6F, H). In addition, CM from overexpressed RCAN1.4 cells reduced the tube formation of HUVECs when compared with the control CM (Fig. 6I). Knockdown RCAN1.4 expression in PDAC cells enhanced HUVECs tube formation, which could be significantly blocked by VEGFA neutralization (Fig. 6J). In conclusion, these results suggested that *VEGFA* as a direct transcriptional target of NFAT1 play a significant role in RCAN1.4-mediated angiogenesis.

**Fig. 7:**
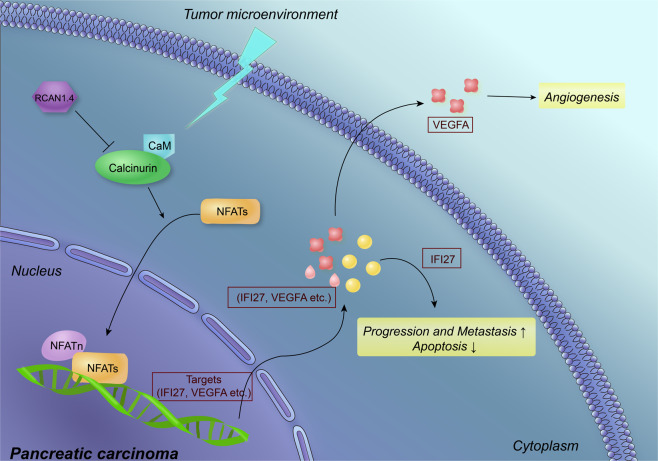
Mechanism map showing a proposed molecular pathway of RCAN1.4-regulated PDAC progression and metastasis. The loss of RCAN1.4 leads to an activation of calcineurin–NFAT1 signaling pathway, which promotes IFI27 and VEGFA expression to promote the malignancy of PDAC.

Finally, schematic diagram of RCAN1.4-mediated PDAC progression of major molecular pathway was presented (Fig. 7). In PDAC cells, the loss of RCAN1.4 leads to an activation of calcineurin–NFAT1 signaling pathway, which promotes IFI27 and VEGFA expression to promote the malignancy of PDAC.

## Discussion

Although RCAN1.4 has been reported in other tumors, its role of PDAC was unknown. In the present study, we determined several aspects of the function and mechanism of RCAN1.4 in PDAC. RCAN1.4 expression was low in PDAC tissues compared with that in its para-cancerous tissues, and PDAC patients with low levels of the RCAN1.4 protein had a shorter OS and more vascular invasion compared with those with high RCAN1.4 levels, which was the same as the results for RCAN1.4 in HCC, RCC, and thyroid cancer [17–19]. Furthermore, we have revealed that patients with low serum RCAN1 level had a shortened OS compared to those with high level. Statistical analysis revealed that low serum RCAN1 level was associated significantly with vascular invasion and a higher serum CA19-9 level. Taken together, we can confirm that the RCAN1.4 protein level can serve as an independent prognostic indicator of PDAC by Cox regression analysis. In our study, our results can provide possible diagnostic criteria and therapeutic targets for PDAC.

We revealed that RCAN1.4 regulates tumor growth, apoptosis, migration, invasion, and angiogenesis in PDAC cell lines in vitro. These results were conformed in vivo, which showed that RCAN1.4 could modulate tumor growth by regulating tumor apoptosis and tumor angiogenesis. Unexpectedly, the levels of Ki-67, a tumor proliferation biomarker, showed no significant differences in the subcutaneous xenograft tumors. It may be that there are many differences between in vitro 2D cell culture and in vivo cell growth conditions. Another reason may be that when the tumors were sacrificed at the endpoint, the proliferation rate of the tumor cells in the large size group decreased, leading to an offset of the differences in proliferation between the two groups, caused by the reduction in tumor growth space. Our data demonstrated a significant increase in the microvessel number in RCAN1.4 KD-BxPC-3 tumors, and RCAN1.4 OE-SW1990 tumors showed the opposite result. This result is supported by several studies that demonstrated that RCAN1.4 plays a vital role in regulating VEGF-mediated tumor angiogenesis by inhibition of the calcineurin pathway in vascular endothelium cells [10–13]. In addition, we demonstrated that RCAN1.4 was positive correlation with most immune effector cells and immune effector factors in PDAC using datasets from the TCGA database. Interesting, most immunoinhibitors and MHC molecules also significantly positive with RCAN1.4 in PDAC (Supplementary Fig. 8A–D). In addition, we first found RCAN1.4 overexpression cell showed significantly weakened resistance to T-cell-mediated tumor cell-killing in vitro (Supplementary Fig. 9A, B). Our results are the first time to report the correlation between RCAN1.4 and tumor immune environment, especially T cells. This conclusion not only implied that RCAN1.4 affects the biological function of tumor cells, but also plays a vital role in the interaction between other components in the tumor microenvironment and these tumor cells. This interaction will be explored later.

Previous studies focused mainly on RCAN1.4’s role in regulating the calcineurin/NFAT pathway in multiple cancers, and showed that targets of NFAT pathway are mainly cytokines, such as VEGF, IGF1, IL-2, and IL-4 [10, 17]. However, we recognized IFI27 as an RCAN1.4 downstream functional effector whose expression correlated negatively with that of RCAN1.4. Importantly, we find that NFAT1 increasingly bound potential binding sites in the IFI27 and VEGFA promotor in PDAC cells by ChIP assay. Although Wang’s group identified that NFE2L3 is a major downstream regulator in RCAN1.4-mediated tumorigenicity and invasion, IFI27 was also one of the most overexpressed genes in FTC236 shRCAN1.4 cells in the RNA-seq data, as shown in Supplementary Fig. S3 [18]. IFI27 is a hydrophobic mitochondrial protein of 122 amino acids that participates in many biological processes, including apoptosis and innate immunity, which maintains a low expression level in multiple mammalian cells, while it is highly expressed in uterine fibroids and certain cancers [20–22]. Indeed, expression analysis of *IFI27* at the TCGA database in our study demonstrated that *IFI27* is overexpressed in 24 types of cancer. In addition, IFI27 is strongly and positively associated with tumorigenesis genes. These results suggested that IFI27 has a broad function across cancer types. For instance, high expression of IFI27 can promote tumor cell proliferation, invasion, and reduced apoptosis in HCC, gastric cancer, and oral squamous cell carcinoma [23–25]. However, IFI27’s role in PDAC is poorly understood. In the present study, IFI27 expression was increased in PDAC tissue, which was associated significantly with poor clinical outcomes, such as OS, stage, and grade of PDAC, as indicated by the TCGA database analysis. Importantly, we provided evidence that overexpression of *IFI27* could promote the invasion, migration, and proliferation of PDAC cells and could revert the promotion of migration and invasion by RCAN1.4 KD in PDAC tumor cells. Therefore, our findings revealed important potential biological roles of RCAN1.4-calcineurin–NFAT1-IFI27 pathway in PDAC, which require further study.

In conclusion, we provided evidence that in PDAC, RCAN1.4 regulates tumor growth, apoptosis, metastasis, and angiogenesis both in vitro and in vivo. The detailed mechanism by which RCAN1.4 exerts these effects involves calcineurin–NFAT1-IFI27/VEGFA, is a crucial regulator in PDAC. This warrants further study of RCAN1.4 and IFI27 as a potential regulatory axis of cancer progression, and targeting this pathway might be a promising therapeutic option for the clinical management of PDAC.

## Materials and methods

### Tissue samples, patients’ serum, and clinical information

Ten paired samples of human PDAC primary tumors and adjacent normal tissues were obtained from patients at the First Affiliated Hospital, Zhejiang University School of Medicine. After resection, all the samples were placed in liquid nitrogen and stored at −80 °C before RNA and protein extraction. Evaluation of *RCAN1.4* expression was carried out on tissue microarrays from 154 patients by IHC using RCAN1.4 antibody (Sigma-Aldrich, 1:100 dilution, catalog D6694). Blood samples were collected from patients with PDAC before operation, separated from serum, and stored at −80 °C. Baseline clinical data, including gender, age, CA19-9, CA125, tumor size, tumor grade, and clinical TNM staging, were collected retrospectively. Regular follow-up was performed and the survival time of the patients was generally defined as the duration between the operation of curative resection and death. All patients provided written informed consent, and the Medical Ethics Review Committee of the First Affiliated Hospital of Zhejiang University School of Medicine approved the study.

### Western blotting and antibodies

Radioimmunoprecipitation assay buffer was used to extract proteins from cells and patient tissues, which were then subjected to sodium dodecyl sulfate-polyacrylamide gel electrophoresis for separation, followed by transfer onto polyvinylidene fluoride (PVDF) membranes (Bio-Rad, Hercules, CA, USA). The proteins on the PVDF membranes were blocked using 10% skim milk for 1 h at room temperature, the membrane was washed with Tris-buffered saline with Tween 20 (TBST), and then incubated with primary antibodies at 4 °C overnight. The membrane was then washed with TBST and incubated with corresponding species second antibodies for 4 h at 4 °C.

α-tubulin glyceraldehyde-3-phosphate dehydrogenase (GAPDH) and Histone H1.2 were used as internal controls. The signal from the immunoreactive proteins were detected using an EzWay DAB Western Blot Kit (KOMA BIOTECH). Abcam (Cambridge, MA, USA) proved the primary antibodies recognizing RCAN1 (1:1000 dilution, catalog ab185931), GAPDH (1:1000 dilution, catalog ab8245), VEGFA (1:1000 dilution, catalog ab46154), NFAT1 (1:1000 dilution, catalog ab2722), and NFAT3 (1:1000 dilution, catalog ab99431). Interferon alpha inducible protein 27 (IFI27) (1:1000 dilution, catalog MBS540037) antibodies were purchased from MyBioSource. NFAT2 (1:1000 dilution, catalog 66963-1-Ig), NFAT4 (1:1000 dilution, catalog 18222-1-AP), Histone H1.2 (1:1000 dilution, catalog 19649-1-AP), CaN (1:1000 dilution, catalog 13340-1-AP) antibodies were purchased from Proteintech.

### RNA isolation and quantitative real-time reverse transcription PCR (qRT-PCR)

Total RNA was extracted from tissues or cell using the Trizol LS Reagent (Invitrogen) and reverse transcribed into cDNA using a PrimeScript RT reagent Kit (Takara, Osaka, Japan). Quantitative real-time PCR analysis was performed in a 20-µl reaction using the SYBR Premix Ex TaqTM II (Takara) in an Applied Biosystems 7500 Fast Real-Time PCR System (Applied Biosystems, Foster City, CA, USA). Relative gene expression was normalized to *GAPDH* expression and was calculated using the standard 2^−^^ΔΔCt^ method. All primers were synthesized by SunYa Inc. (Hangzhou, China) (Supplementary Table 1).

### Cell invasion and migration assays

Twenty-four-well Matrigel-uncoated chambers with 8-μm pore membranes (Corning Life Sciences, cat. no. 353097) were used to assess the cells’ migration ability. Cells (PANC-1/MIA PaCa-2/BxPC-3: 5 × 10^5^/well; HUVEC: 1 × 10^5^/well; SW1990: 1 × 10^6^/well) were placed onto the upper chamber in serum-free medium and as a chemoattractant, 500 μl of medium with 10% FBS was placed into the lower chamber. Then, the cells in the chambers were incubated in 5% CO_2_ at 37 °C. Cells that migrated were fixed using 10% formalin and stained with crystal violet (0.1%). The number of migratory cells was determined using the Image-Pro Plus 6.0 image analysis software (Media Cybernetics, Bethesda, MA, USA). Determination of each sample was conducted in quintuplicate. Assays of cell invasion were performed in Matrigel-coated (1 mg/ml Matrigel matrix, BD Biosciences) Transwell insert chambers (Corning Life Sciences, cat. no. 353097). The other steps followed the same procedures as described above for the migration assay.

### Animal model

The ethical guidelines established by the Ethics Committee of hospital were followed during the animal experiments. Cells (1 × 10^6^) were collected by centrifugation and suspended in 100 μl of serum-free medium. Mice were purchased from the model animal research center of Nanjing University and were 6 weeks old. For orthotopic injection model, the cells suspension was injected into the pancreas of athymic nude mice. RCAN1.4 overexpression BxPC-3 cells and relative control cells, respectively, injected into the pancreas of seven athymic nude mice. RCAN1.4 knockdown SW1990 cells and control cells, respectively, injected into the pancreas of five athymic nude mice. After 1 month, the mice were euthanized and the tumor was carefully detached. The tumor weight was determined, and then the tumor was stored in 10% neutral buffered formalin. For liver metastasis model, the cells suspension was injected into the spleen of athymic nude mice. RCAN1.4 knockdown SW1990 cells, RCAN1.4 overexpression PANC-1 cells and their control cells, respectively, injected into the spleen of five athymic nude mice. After 1 month, the mice were euthanized and the liver was carefully detached.

### Histology and immunohistochemistry (IHC)

Tumors and tissues were fixed with 10% neutral buffered formalin, embedded in paraffin and cut into slices. IHC was then performed using antibodies recognizing RCAN1 (1:100 dilution, Sigma-Aldrich, catalog D6694), CD31 (1:250 dilution, Santa Cruz, catalog sc-1506R), cleaved caspase-3 (1:200 dilution, Cell Signaling, catalog 9661), ki67 (1:100 dilution, Abcam, catalog ab15580), and IFI27(1:200 dilution, Abcam, catalog ab224133) for mouse tumors harvested at the end of the experiment.

Each slide was assessed in five high-power field images (×100) using the Vectra imaging system (Perkin Elmer). GraphPad Prism 8 (GraphPad Software, San Diego, CA, USA) was used to quantify the IHC staining. Wuhan Servicebio technology performed IHC staining of the tissue array. The IHC results were quantified by processing the images using 3D HISTECH quant center 2.1 software.

### RNA sequencing

Total RNA of RCAN1.4-BxPC-3 and WT-BxPC-3 was extracted and converted into cDNA libraries for RNA-seq analysis using the IlluminaHiseq sequencing platform supported by Novogene (Beijing, China). Data of RNA sequences deposited in Sequence Read Aechive (accession codes: SAMN17373567, SAMN17373568).

### Chromatin immunoprecipitation assay

PDAC cells of RCAN1.4 knockdown and the relative control cells were cultured at about 80–90% confluency in 10 cm Petri dish. According to the manufacturer’s instruction, ChIP assay was performed using the ChIP Assay Kit (P2078; Beyotime, China). The cells were cross-linked with 1% formaldehyde at 37 °C for 10 min, and then neutralized with glycine for 5 min. Cold PBS with 1 mM PMSF washing three times, the cells were harvested and sonicated in SDS lysis buffer. After centrifugation, supernatants were collected and diluted with ChIP dilution buffer. One in ten of the total liquid was used as input. The rest of liquid was added into primary antibody to immunoprecipitate cross-linked protein-DNA complexes. The immunoprecipitated DNA was purified and extracted using DNA extraction kit. The results were detected by PCR. All Chip primers were synthesized by Sangon Inc. (Shanghai, China) (Supplementary Table 2).

### The Cancer Genome Atlas data analysis

The Cancer Genome Atlas gene expression RNA sequencing data for analysis of RCAN1.4 and IFI27 were downloaded using two web portals: Tumor and Immune System Interaction Database (http://cis.hku.hk/TISIDB) and Gene Expression Profiling Interactive Analysis (GEPIA2, http://gepia2.cancer-pku.cn).

### Statistical analyses

Student’s *t* test was used to analyze continuous variables for two groups. The chi-squared test was used to analyze the categorical variables. To analyze the correlation between survival and the expression of RCAN1.4, Kaplan–Meier curves with log-rank tests were constructed. Univariate and multivariate Cox proportional hazard regression analyses were used to estimate the prognostic significance of RCAN1.4 in PDAC. A two-tailed *p* value <0.05 was considered statistically significant. SPSS Statistics 22.0 (IBM Corp., Armonk, NY, USA) and GraphPad Prism 6 were used to perform the statistical analyses.

## Supplementary information

Supplementary Materials and Methods

Supplementary figure and table legends

Supplementary figure 1

Supplementary figure 2

Supplementary figure 3

Supplementary figure 4

Supplementary figure 5

Supplementary figure 6

Supplementary figure 7

Supplementary figure 8

Supplementary figure 9

Supplementary table 1

Supplementary table 2
