# Supplementary material for: Regulator of calcineurin 1 gene isoform 4 in pancreatic ductal adenocarcinoma regulates the progression of tumor cells
Source: Oncogene. 2021 Apr 6;40(17):3136–51. doi: 10.1038/s41388-021-01763-z (PMC8084734; doi:10.1038/s41388-021-01763-z)
Supplement: Supplementary file 1 — Supplementary Materials and Methods [file 41388_2021_1763_MOESM1_ESM.docx]

**Supplementary Materials and Methods**

**1. Cell culture.**

The American Type Culture Collection (ATCC; Manassas, VA, USA) provided the PDAC cell lines PANC-1, T3M4, BxPC-3, SW1990, MIAPaCa-2, and HPNE. Human pancreatic duct epithelial (HPDE) cells were purchased from the Kerafast company (Boston, MA, USA). Pancreatic stellate cells (PSCs) and Human umbilical vein endothelial cell (HUVEC) were obtained from the Sciencell company. Roswell Park Memorial Institute (RPMI)-1640 medium (Gibco, Thermo Fisher Scientific, Waltham, MA, USA) was used to culture PANC-1, BxPC3, T3M4, and SW1990 cells. Dulbecco’s Modified Eagle’s medium (DMEM) (Gibco) was used to culture MIAPaCa-2 cells. HPNE cells were cultured in 75% DMEM plus 25% Medium M3 Base. HPDE cells were cultured in Keratinocyte SFM, + EGF + bovine pituitary extract (Invitrogen, Carlsbad, CA, USA, Cat#:17005042). PSCs were cultured in Stellate Cell Medium (Sciencell, Cat#:5301). 10% fetal bovine serum (FBS; Thermo Fisher Scientific) and 1% penicillin-streptomycin (Genom, Hangzhou, China) were used to supplement all culture media. An additional 2.5% (final concentration) horse serum (Gibco) was required to culture MIAPaCa-2 cells. All cells were cultured in a humidified incubator at 37 °C with 5% CO_2_.

**2. Stable cell line generation**

BxPC-3 and PANC-1 cells in good condition were seeded at 1 × 10^4^ cells/mL into a 24-well plate. When the cells had completely adhered to the wall, the medium was replaced with 1 mL of a serum-free medium. The cells in the two wells were then added with 20 μL RCAN1.4 overexpression or negative control lentivirus (Oobio, Shanghai, China) with sufficient mixing, respectively. Approximately 16–24 h after transduction, serum-containing medium replaced the serum-free medium and incubation of the cells was continued for 48 h. Puromycin (2 μg/mL; Sigma-Aldrich, St. Louis, MO, USA) was used to select the overgrown cells for 3 days in a 6-well plate. Transduction efficiency was determined by the expression of the green fluorescence protein (GFP) under an inverted fluorescence microscope (Leica, Wetzlar, Germany). The overexpression of RCAN1.4 in the cell lines was screened using real-time PCR and western blotting. Thus, we obtained cell lines that stably overexpressed RCAN1 and those containing the negative control vector. The MIAPaCa-2 and SW1990 cells with knockdown of *RCAN1.4* and the negative control vector were obtained in the same way.

**3. Cell proliferation**

Cell counting kit-8 (CCK-8; Dojindo, Kumamoto, Japan) and 5-Ethynyl-2´-deoxyuridine (EdU) (Invitrogen) assays were performed to detect cell proliferation *in vitro*. PDAC cells were seeded in 96-well plates. When the density of cells per well reached 70%, each well was washed with cold phosphate-buffered saline (PBS), and added with serum-free medium containing 10% CCK-8 up to a final volume of 100 μl. After incubation for at 37 °C 2­–4 h, the absorbance at 450 nm was determined. According to manufacturer's protocol, the cells were stained with EdU using an EdU Kit (Ribobio, Beijing, China) in 96-well plates. The results were analyzed using fluorescence microscopy (Leica DMI4000B).

**4. Nuclear protein extraction**

NE-PER™ Nuclear and Cytoplasmic Extraction Reagents (Thermo Scientific, catalog 78833, USA) was used to segregate the cytoplasm and nuclear protein. The Histone H1.2 and GAPDH as the references of nuclear and cytoplasm protein, respectively.

**5. Determining Apoptosis**

Flow cytometry with the Annexin V–FITC–PI Kit (BD Pharmingen, San Diego, CA, USA) was used to detect the cell apoptosis rate. Cells grown in 6-well tissue culture plates were collected and stained using the Annexin V–FITC–PI Kit, based on the manufacturer's instructions. Flow cytometry using a FACS Caliber flow cytometer (Becton Dickinson, San Jose, CA, USA) was used to analyze the differences between RCAN1.4 overexpression BxPC-3/PANC-1 and control group.

**6. Immunofluorescence**

PDAC cells of RCAN1.4 modulation and the relative control cells were cultured at about 80–90% confluency in 12-well plates, then fixed with 4% paraformaldehyde for 5 min at room temperature, permeabilized with 0.1% Triton X-100 for 15min, blocked with 1% bovine serum albumin (BSA) for 30 min. After incubation with the primary antibody at 4 °C overnight, three washed with PBS, the cells were incubated with 1% Alexa flour 555 second antibody (Invitrogen) for 1 h at 37℃. Nuclei were stained with 0.02μg/mL Hoechst (Servicebio) for 2 min. Three washed with PBS, the cells were imaged by confocal laser scanning microscopy TCS SP8 CARS (Leica, Germany).

**7. Subcutaneous injection model.**

Cells (1 × 10^6^) were collected by centrifugation and suspended in 100 μl of serum-free medium. Mice were purchased from the model animal research center of Nanjing University and were 6 weeks old. The cells suspension was injected into the ventrolateral flank of athymic nude mice subcutaneously. Vernier calipers were used to measure the tumors weekly. The following formula was used to determine the tumor volume: Tumor volume = 1⁄2 × (length × width × width). After 1 month, the mice were euthanized and the tumor was carefully detached. The tumor weight was determined, and then the tumor was stored in 10% neutral buffered formalin.

**8. Tube Formation Assay on Matrigel**

Matrigel (10 μl; BD Bioscience) was casted into each well of a μ-Slide Angiogenesis (iBidi, Munich, Germany) and allowed to solidify at 37℃. For each well, 10,000 cells/well of HUVEC were seeded on the Matrigel and cultured in the incubator. After cell attachment, carefully aspirate the primary medium and replace it by 50 μl cell-free conditioned medium. Visualization and image acquisition were performed using an inverted microscope. The program Image J was used to measure the number of tubulars.
