# Supplementary material for: Regulator of calcineurin 1 gene isoform 4 in pancreatic ductal adenocarcinoma regulates the progression of tumor cells
Source: Oncogene. 2021 Apr 6;40(17):3136–51. doi: 10.1038/s41388-021-01763-z (PMC8084734; doi:10.1038/s41388-021-01763-z)
Supplement: Supplementary file 2 — Supplementary figure and table legends [file 41388_2021_1763_MOESM2_ESM.docx]

**Supplementary figure and table legends**

**Figure S1.** (A) Correlation between the mRNA levels of RCAN1.4 and tumor grade in PAAD patients. (B) Correlation between the mRNA levels of RCAN1.4 and tumor stage in PAAD patients. (C) The Cox regression analysis of OS based on RCAN1.4 protein levels in PDAC patients was performed.

**Figure S2.** **RCAN1.4 regulates pancreatic cancer cell proliferation, migration, and invasion.**

(A) Effects of RCAN1.4 overexpression and knockdown on proliferation of PANC-1 (top) and MIA PaCa-2 (bottom) cells by CCK-8 assay. (B) Representative images and quantification of Edu in RCAN1.4-overexpression BXPC-3 and PANC-1 cells, and RCAN1.4-knockdown MIA PaCa-2 to detect cell viability. (C) Representative images and quantification of migration and invasion of RCAN1.4-overexpression PANC-1 cells. Scale bar = 100 μm. (D) Representative images and quantification of migration and invasion of RCAN1.4-knockdown MIA PaCa-2 cells. Scale bar = 100 μm. Results are presented as mean ± SD from one representative experiment. Error bars, ± SD (determined using a two-tailed t‑test, ns: no significance, *P < 0.05, **P < 0.01, ***P < 0.001, ****P < 0.0001)

**Figure S3.** ***In vivo*, RCAN1.4 shows tumor suppressor functions in PDAC.**

(A-C) Tumor representative images, tumor weight, and growth curve of RCAN1.4‑overexpression BxPC-3 and PANC-1, and RCAN1.4-knockdown SW1990 cells of subcutaneous tumor model in nude mice. Results are presented as mean ± SD from one representative experiment. Error bars, ± SD (determined using a two-tailed t‑test, ns: no significance, *P < 0.05, **P < 0.01, ***P < 0.001, ****P < 0.0001)

**Figure S4.** (A-B) Representative images and quantification of Ki67 staining for the BxPC-3 and SW1990 tumors. Results are presented as mean ± SD from one representative experiment.

**Figure S5.** Cytoplasmic levels of NFAT subtypes (NFAT1-4) were detected by western blot after overexpression or knockdown of RCAN1.4.

**Figure S6.** Confirmation the efficiency after transfecting with calcineurin siRNAs (siCaN#1, siCaN#2, or siRCAN1.4#3) (A) or NFAT1 siRNAs (B) in PDAC cells using immunoblotting analysis.

**Figure S7. IFI27 was confirmed from RNA-seq.**

(A) The differentially expressed genes in the RNA-seq data between RCAN1.4 OE‑BxPC-3 and WT-BxPC-3 cells were validated using qRT-PCR. The top five upregulated genes and top five downregulated genes were chosen. (B) Ten no significantly expressed genes in the RNA-seq data between RCAN1.4 OE‑BxPC-3 and WT-BxPC-3 cells were validated using qRT-PCR. (C) The differentially expressed genes validated by qRT-PCR from the RNA-seq data were confirmed using western blotting. OE, overexpression (D) IFI27 levels and their association with OS was determined using Kaplan–Meier analysis. Samples from 103 patients with PAAD were subjected to IFI27 IHC staining. IFI27 levels and their association with OS was determined using Kaplan–Meier analysis in a PDAC tissue microarray.

**Figure S8.** **Analyzation of association between RCAN1.4 and immune factors in PAAD using TCGA database.**

(A) Heatmap visualization of the correlation between *RCAN1.4* and immune effector cells in multiple cancer types using datasets from the TCGA database. (B) Bioinformatic analysis of the correlation between *RCAN1.4* and *CD3E/CD4/CD8A/GZMB* in pancreatic cancer using datasets from the TCGA database. TPM, transcripts per million (C-D) Bioinformatic analysis of the correlation between *RCAN1.4* and immunoinhibitors and MHC molecules in pancreatic cancer using datasets from the TCGA database. TPM, transcripts per million

**Figure S9.** **The role of RCAN1.4 on the tumor immunity in PDAC**

(A) Immunoblot in KPC cells stably overexpressing RCAN1.4. (B) Representative images and quantification of T cells-killing assay was performed in RCAN1.4-overexpression KPC cells. Results are presented as mean ± SD from one representative experiment. Error bars, ± SD (determined using a two-tailed t‑test, ns: no significance, *P < 0.05, **P < 0.01, ***P < 0.001, ****P < 0.0001)

**Table S1.** **Sequences of Real-Time PCR Primers.**

**Table S2. Sequences of CHIP Primers.**
