# Supplementary figures and images for: Regulator of calcineurin 1 gene isoform 4 in pancreatic ductal adenocarcinoma regulates the progression of tumor cells
Source: Oncogene. 2021 Apr 6;40(17):3136–51. doi: 10.1038/s41388-021-01763-z (PMC8084734; doi:10.1038/s41388-021-01763-z)

A

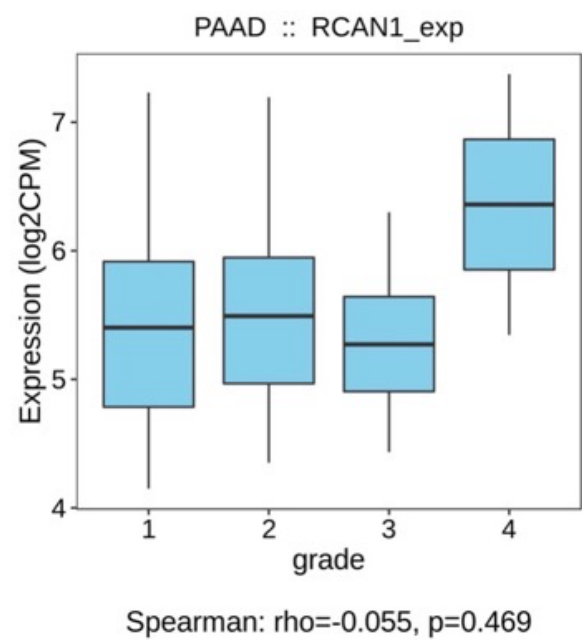

B

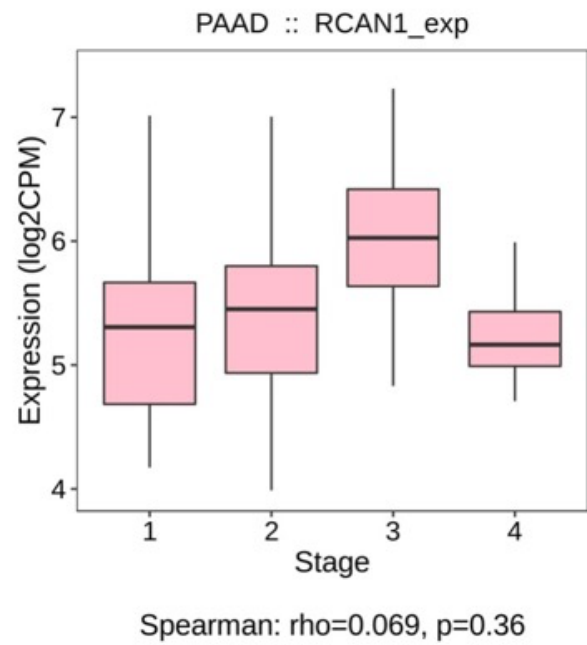

C

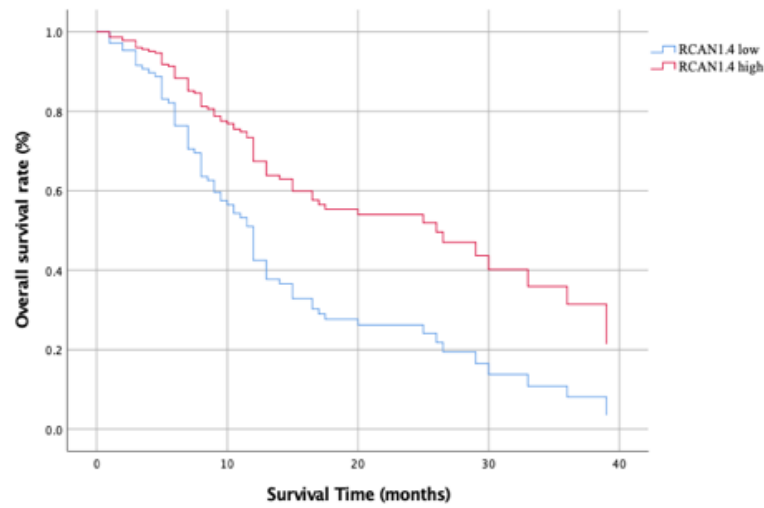

Figure S1

Supplement: Supplementary file 3 — Supplementary figure 1 [file 41388_2021_1763_MOESM3_ESM.pdf]

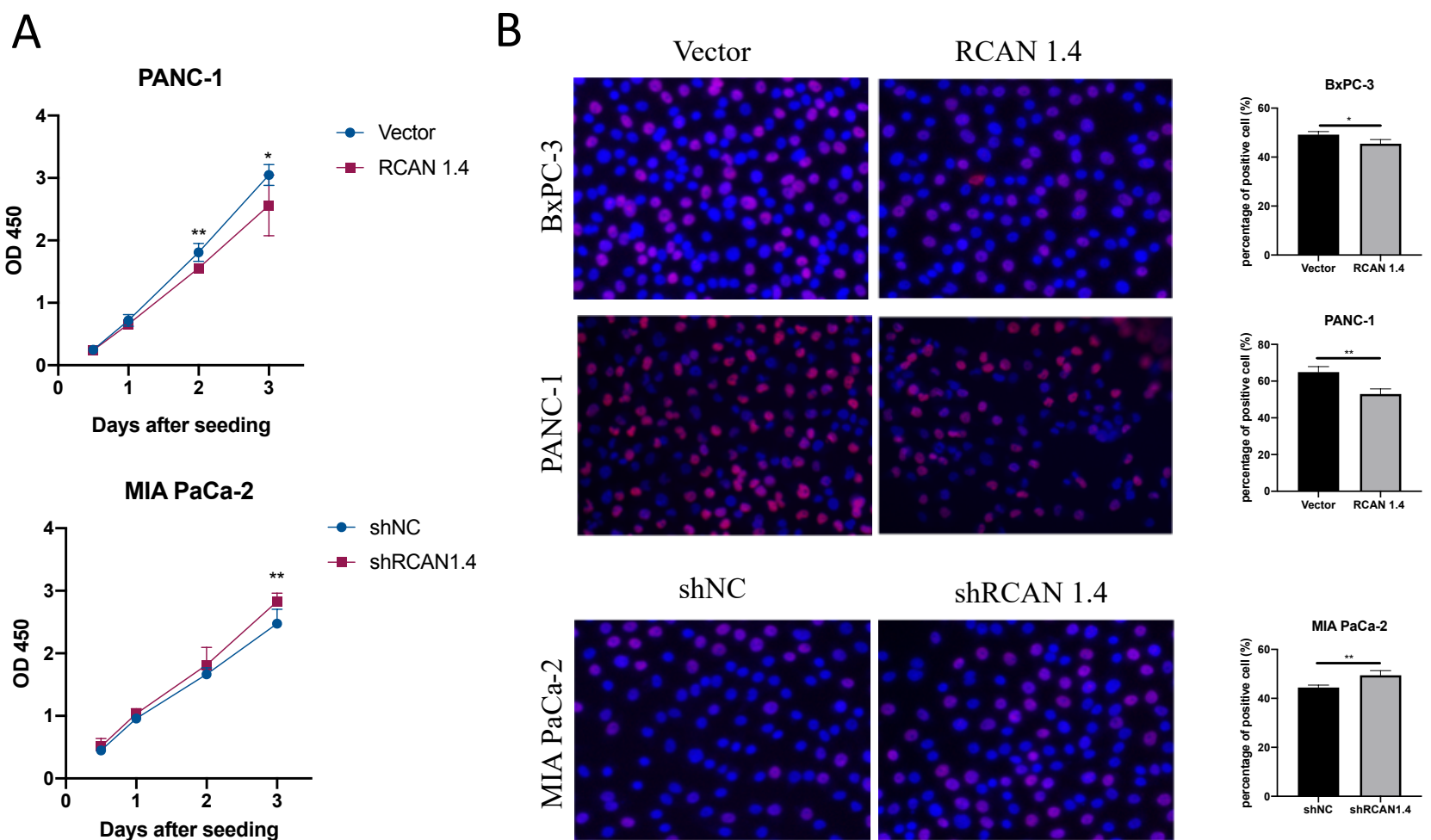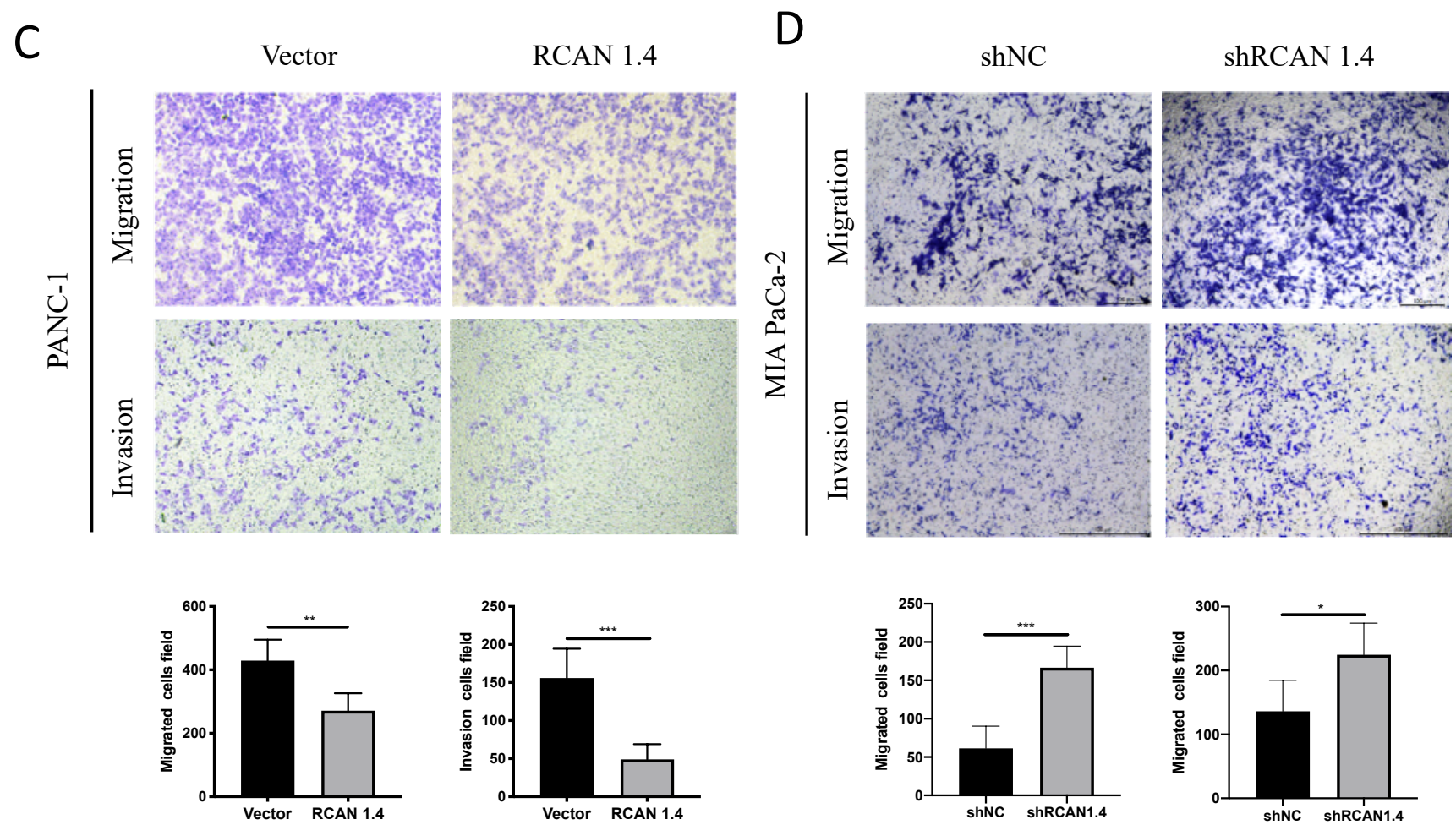

Figure S2

Supplement: Supplementary file 4 — Supplementary figure 2 [file 41388_2021_1763_MOESM4_ESM.pdf]

A

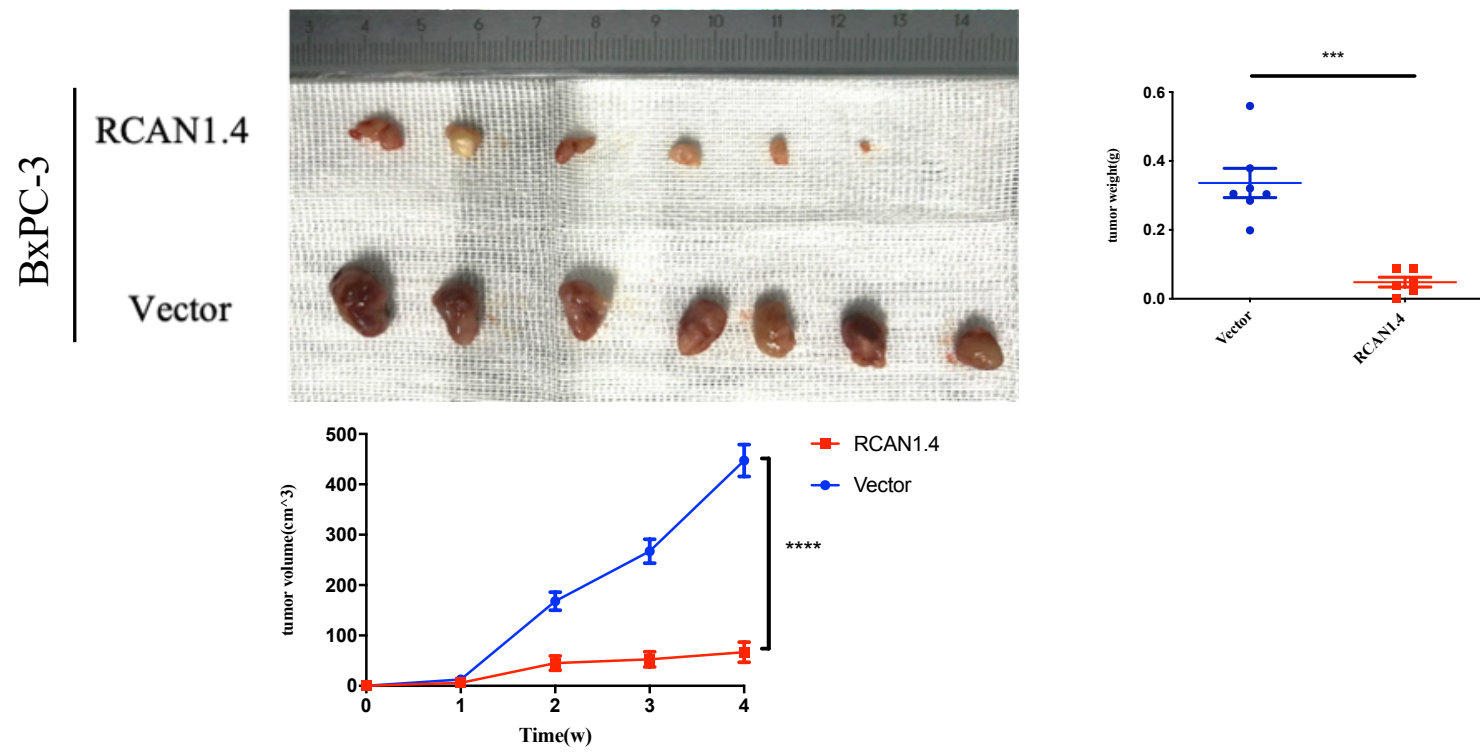

B

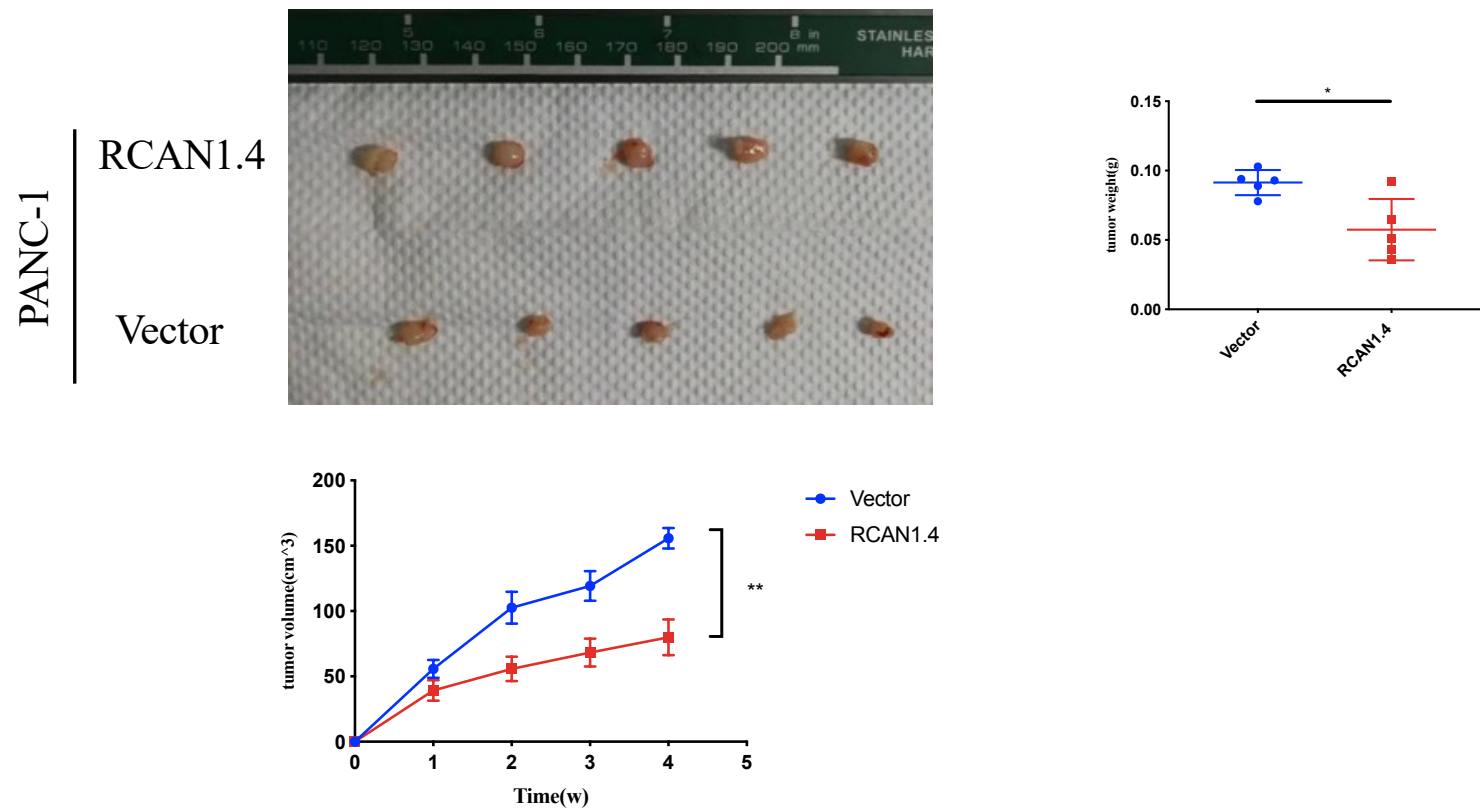

C

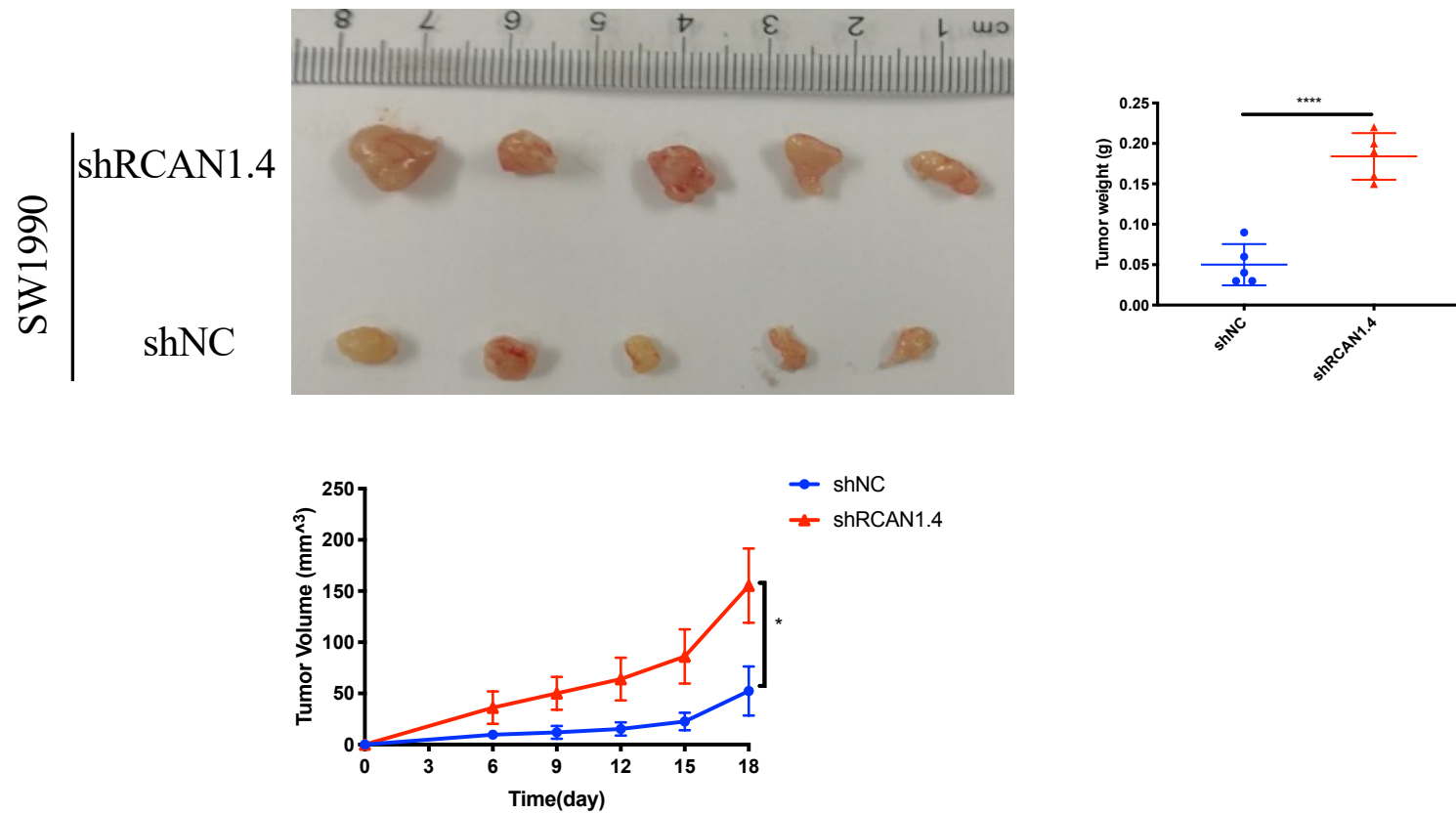

Figure S3

Supplement: Supplementary file 5 — Supplementary figure 3 [file 41388_2021_1763_MOESM5_ESM.pdf]

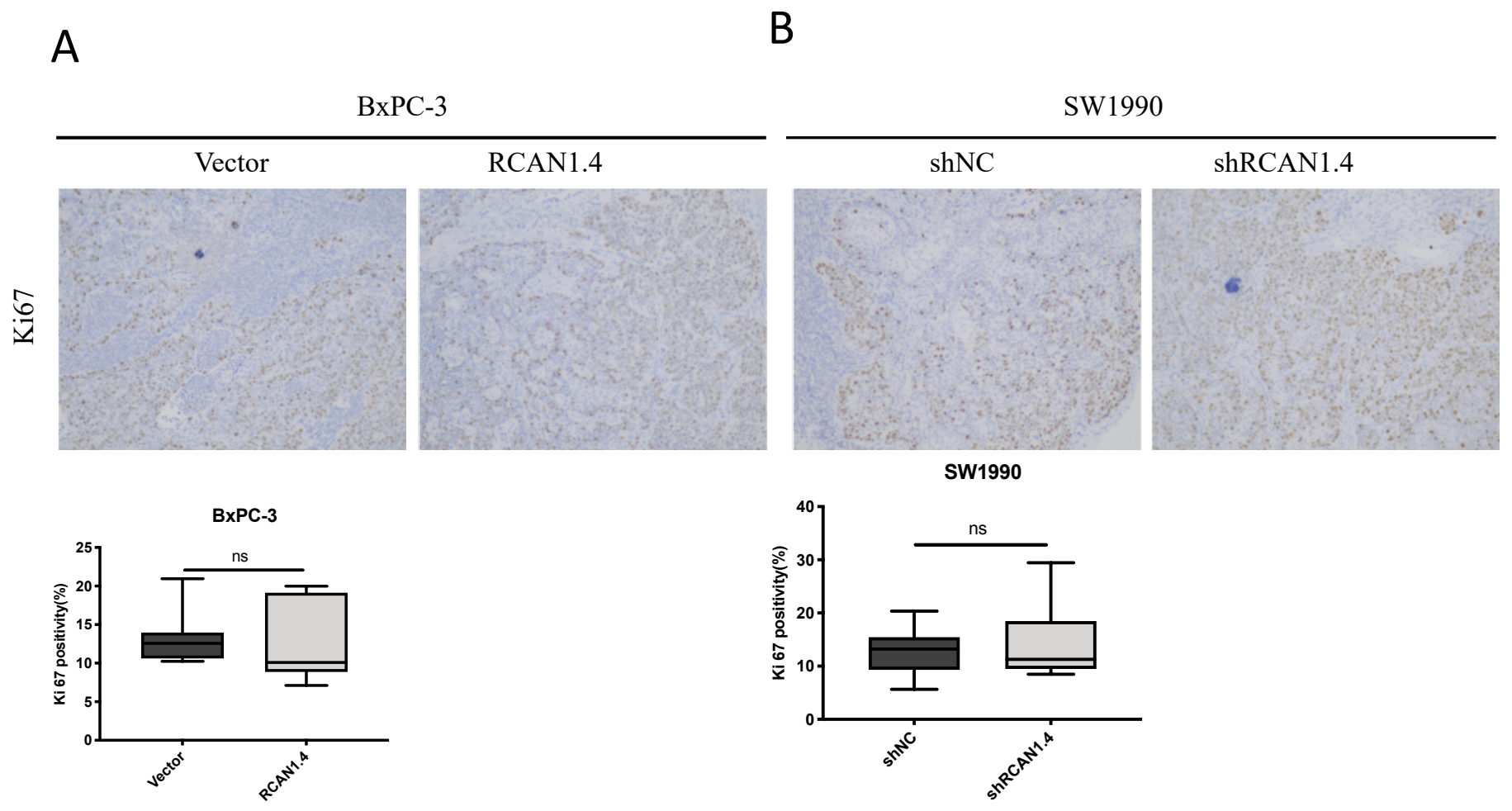

Figure S4

Supplement: Supplementary file 6 — Supplementary figure 4 [file 41388_2021_1763_MOESM6_ESM.pdf]

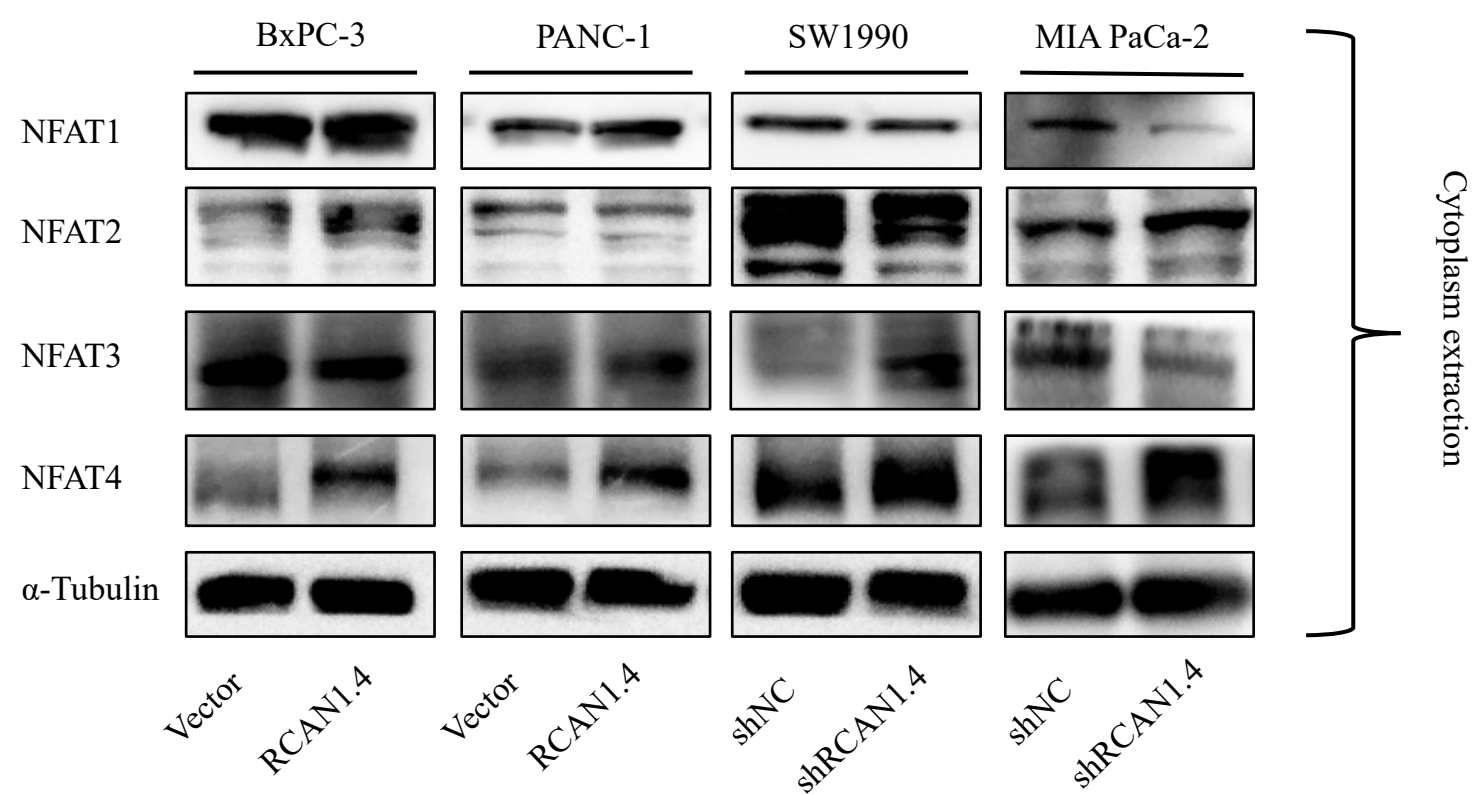

Figure S5

Supplement: Supplementary file 7 — Supplementary figure 5 [file 41388_2021_1763_MOESM7_ESM.pdf]

A

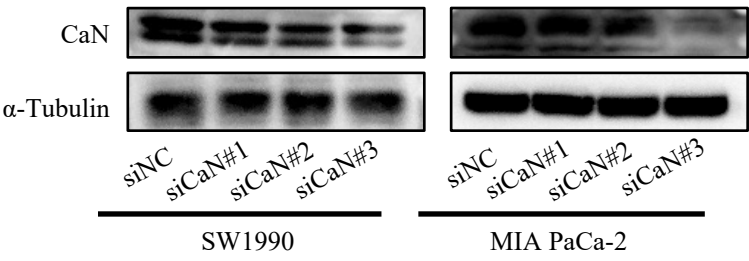

B

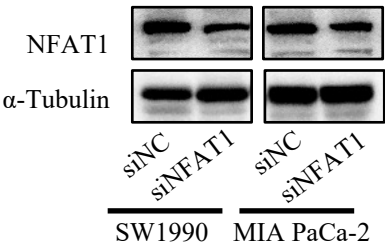

Figure S6

Supplement: Supplementary file 8 — Supplementary figure 6 [file 41388_2021_1763_MOESM8_ESM.pdf]

A

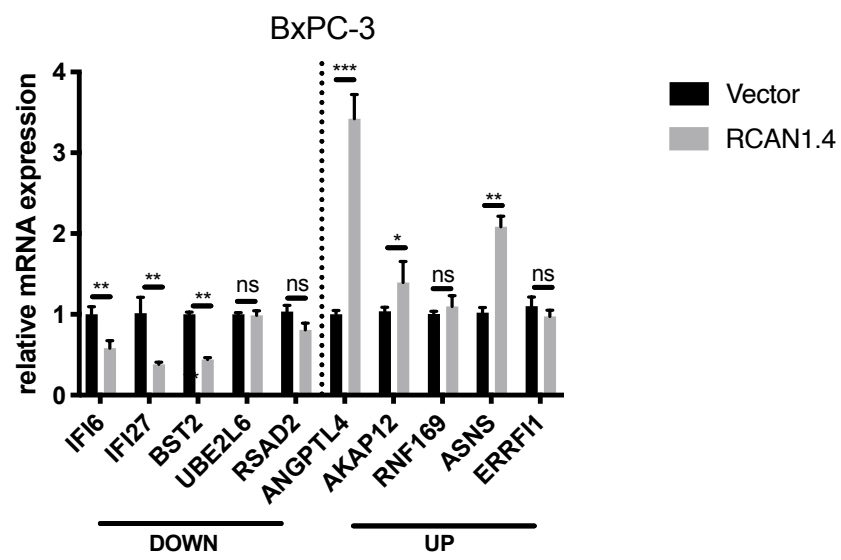

B

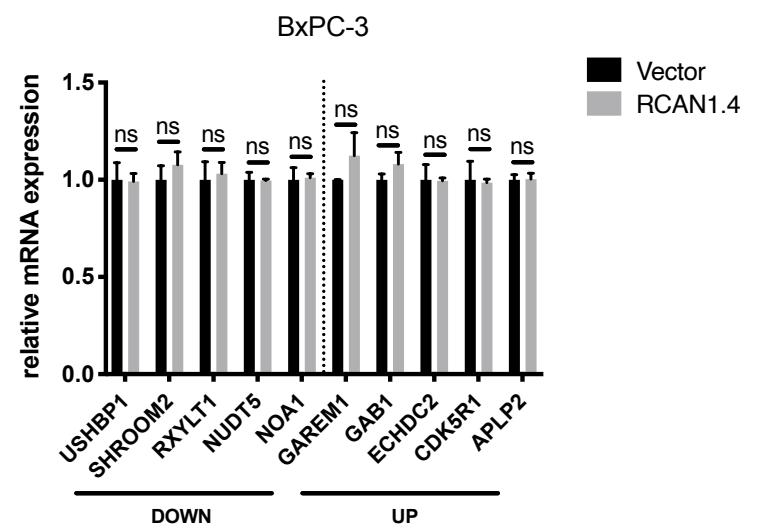

C

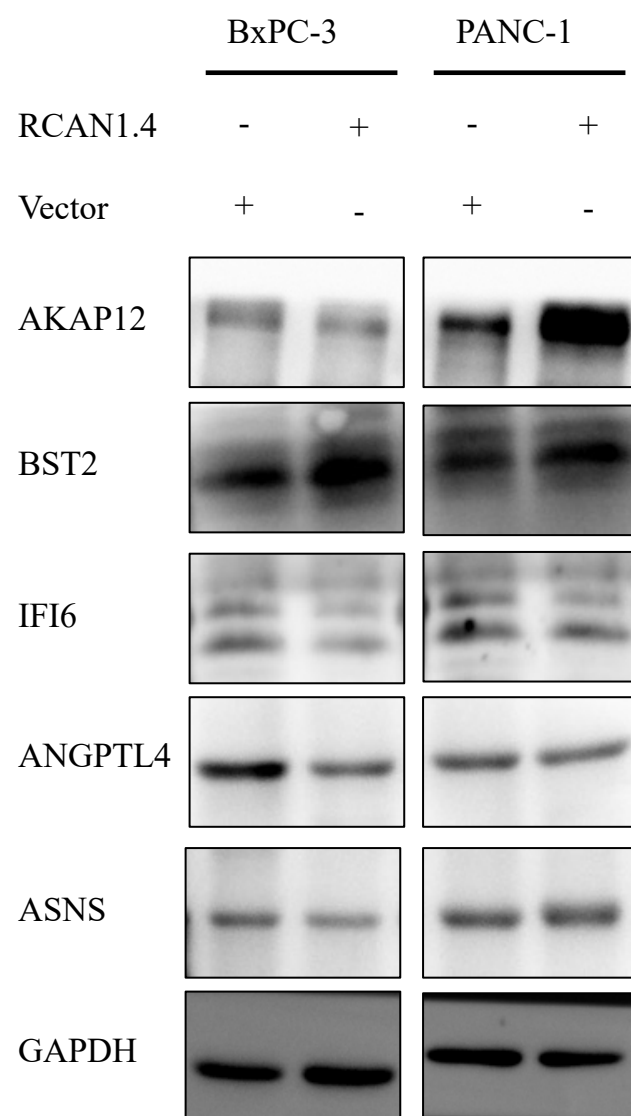

D

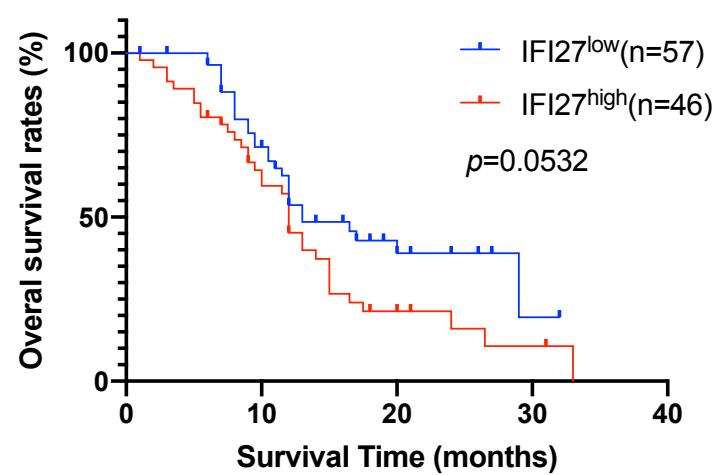

Figure S7

Supplement: Supplementary file 9 — Supplementary figure 7 [file 41388_2021_1763_MOESM9_ESM.pdf]

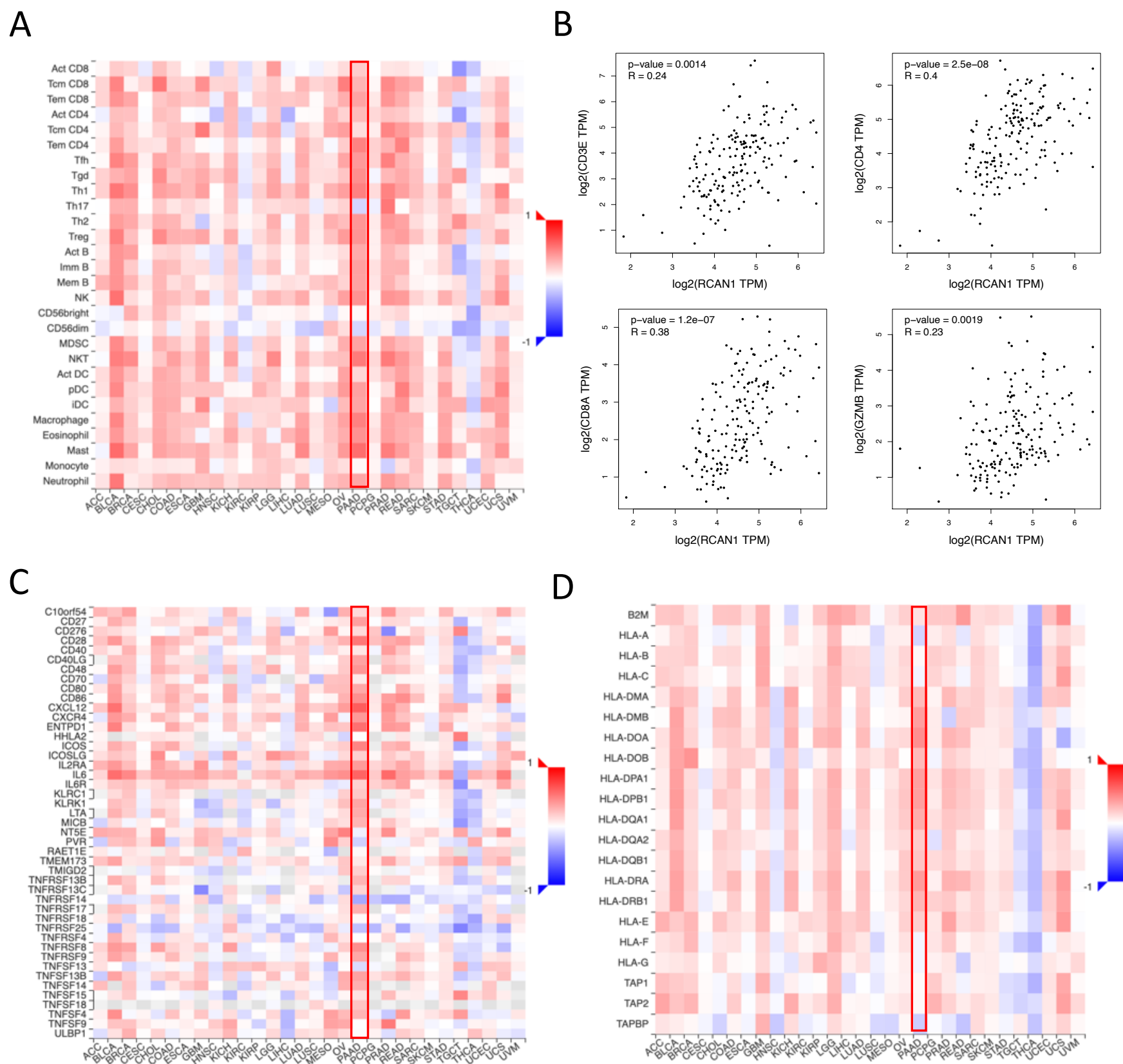

Figure S8

Supplement: Supplementary file 10 — Supplementary figure 8 [file 41388_2021_1763_MOESM10_ESM.pdf]

A

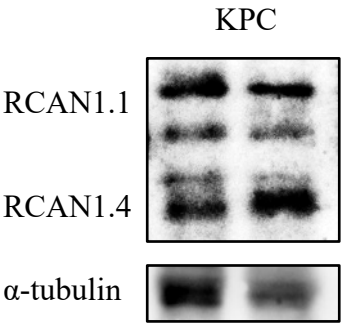

B

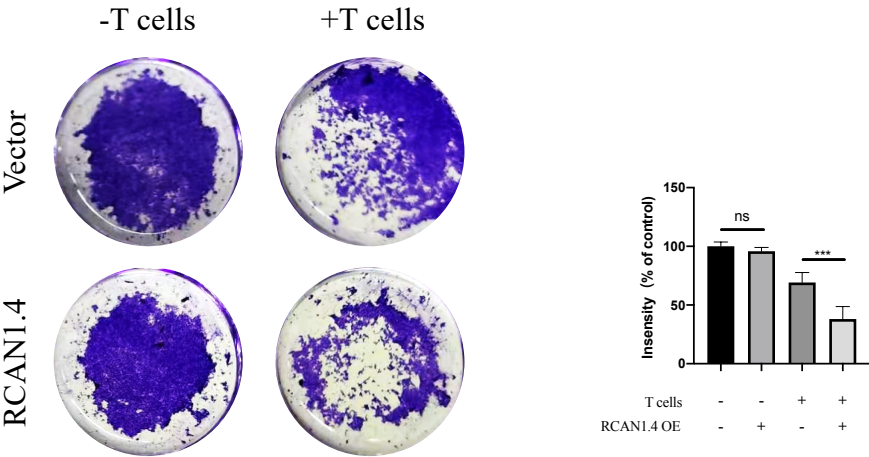

Figure S9

Supplement: Supplementary file 11 — Supplementary figure 9 [file 41388_2021_1763_MOESM11_ESM.pdf]
