# Supplementary material for: Regulator of calcineurin 1 gene isoform 4 in pancreatic ductal adenocarcinoma regulates the progression of tumor cells
Source: Oncogene. 2021 Apr 6;40(17):3136–51. doi: 10.1038/s41388-021-01763-z (PMC8084734; doi:10.1038/s41388-021-01763-z)
Supplement: Supplementary file 12 — Supplementary table 1 [file 41388_2021_1763_MOESM12_ESM.docx]

**Table-S1:** **Sequences of Real-Time PCR Primers**

| Gene | Primer Sequences | |
| --- | --- | --- |
|  | Forward | Reverse |
| *RCAN1.4* | 5’-TTTAGCTCCCTGATTGCCTGT-3’ | 5’-AAAGGTGATGTCCTTGTCATACG-3’ |
| *IFI27* | 5'- TCCTTCTTTGGGTCTGGCTG -3' | 5'- GGCCACAACTCCTCCAATCA-3' |
| *IFI6* | 5'- CTCTTCACTTGCAGTGGGGT -3' | 5'- TGCTGGCTACTCCTCATCCT -3' |
| *BST2* | 5'- ATGGAAGACGGGGATAAGCG -3' | 5'- AGGAGATGGGTGACATTGCG -3' |
| *UBE2L6* | 5'- GAGCATGCGAGTGGTGAAGG-3' | 5'- TTCGAGTTGGCTGCTGGATT-3' |
| *RSAD2* | 5'- TGCTGGGAAGCTCTTGAGT-3' | 5'- CATTGCTCACGATGCTCACG-3' |
| *ANGPTL4* | 5'- TCTCTGGAGGCTGGTGGTTT-3' | 5'- GAAAGGGGGCTTCTCCAGTC-3' |
| *AKAP12* | 5'- GGGCCACCAACTGTCCCTTA-3' | 5'- TCCTGTTTCACTGGTCACGG-3' |
| *RNF169* | 5'- GCAACCGACTCTCCCTTCAA-3' | 5'- AAATGCCGAGCGATGTGTCT-3' |
| *ASNS* | 5'- CACGCTGACCCACTACAAGT-3' | 5'- AGAGGGCTGTGAGTTCTTGC-3' |
| *ERRFI1* | 5'- GGAGCAGTCGCAGTGAGTTT-3' | 5'- GGTCTGAGGTGGAGGAGGAT-3' |
| *h-GAPDH* | 5'- GTTAGGAAAGCCTGCCGGTG-3' | 5'- AGCATCGCCCCACTTGATTT-3' |
| ECHDC2 | 5’-CATTGCGGCTATGGATGGG-3’ | 5’- GATGCAATGTCCACCTCCGT-3’ |
| NUDT5 | 5'- CCGAAGCCAAAGCCAGAGTT-3' | 5'- CTTGCGGGATGGACCTGAAT-3' |
| GAB1 | 5'- AGCCAGAAGGAGCATTGTAGT-3' | 5'- AAGAGCTCCCCATTTGTCCC -3' |
| RXYLT1 | 5'- GCTGCCTACCACGTCTTCTT -3' | 5'- CGGGTAATCCCGGACTTGAG -3' |
| GAREM1 | 5'- GAGCGCTGCTATGGCGT-3' | 5'- TCTACGCACTCTCCGTTGTC-3' |
| NOA1 | 5'- TCTACGCACTCTCCGTTGTC-3' | 5'- GATTCGGATTCTCCCCGTCC-3' |
| CDK5R1 | 5'- CAGATCCAAGGGGGCAGC-3' | 5'- CGACAGCGACTTCTTCAGGT-3' |
| SHROOM2 | 5'- CACTTGCGTGAATGCTGGTC-3' | 5'- TCAGAGGCAAACAGGGGAAC-3' |
| APLP2 | 5'- AGTCCGAGTGTGTGAGCTTG-3' | 5'- ACCTCCATCCGCTCTTTGTG-3' |
| USHBP1 | 5'- CCTTGGGTCTCTTCCCAACC-3' | 5'- CACTGTGGGACACTGGTTCT-3' |
