# Supplementary material for: Regulator of calcineurin 1 gene isoform 4 in pancreatic ductal adenocarcinoma regulates the progression of tumor cells
Source: Oncogene. 2021 Apr 6;40(17):3136–51. doi: 10.1038/s41388-021-01763-z (PMC8084734; doi:10.1038/s41388-021-01763-z)
Supplement: Supplementary file 13 — Supplementary table 2 [file 41388_2021_1763_MOESM13_ESM.docx]

| **Table-S2.** **Sequences of CHIP Primers** | |
| --- | --- |
| **Primers for ChIP in the IFI27 promoter:** | |
| IFI27 binding site 1 sense: | AGTTGCTAGGTAGGACTGGGAATCT |
| IFI27 binding site 1 antisense: | TGGGAGGGCTCTCACGC |
| IFI27 binding site 2 sense: | CTGCGTAGAGCACACTCCC |
| IFI27 binding site 2 antisense: | TCCCAAAGGGTGTGATCCCAG |
| IFI27 binding site 3 sense: | TCTTCCGCCTGTTCTCAAAGCAG |
| IFI27 binding site 3 antisense: | CCGTGTGGCAGGAGCAG |
| IFI27 binding site 4 sense: | AGAGAGAGAGGGAGACAAATCTTTAAG |
| IFI27 binding site 4 antisense: | AGCTTTCGGCTGTGGGATC |
| **Primers for ChIP in the VEGFA promoter:** | |
| VRGFA binding site 1 sense: | CTCTTCCCACAGGCCTCAGA |
| VEGFA binding site 1 antisense: | TTCGTCTCAGCTCCCCCATT |
| VRGFA binding site 2 sense: | TTCCCGTTCTCAGCTCCACA |
| VEGFA binding site 2 antisense: | CTCCATTCACCCAGCTTCCC |
| VRGFA binding site 3 sense: | GCCAAGTGGTGGAGACAGGA |
| VEGFA binding site 3 antisense: | TGTGGGAAGAGTGGGACCAG |
| VRGFA binding site 4 sense: | TTCCTTAGTGCTGGCGGGTA |
| VEGFA binding site 4 antisense: | TTGTGGAGCTGAGAACGGGA |
| VRGFA binding site 5 sense: | CCTCCACAGAGGCTATGCCA |
| VEGFA binding site 5 antisense: | TTCGTCTCAGCTCCCCCATT |
| VRGFA binding site 6 sense: | TCTCAGTCCATGCCTCCACA |
| VEGFA binding site 6 antisense: | TTCCCTCAGGATCCCTTGGC |
| VRGFA binding site 7 sense: | ATCTGGAGCCCTCATCTGGC |
| VEGFA binding site 7 antisense: | GACGTTCCTTAGTGCTGGCG |
| VRGFA binding site 8 sense: | ATCTGTGTGTCCCTCTCCCC |
| VEGFA binding site 8 antisense: | ACCCCCACCAAGGTTCACA |
| VRGFA binding site 9 sense: | AGGGAAGCTGGGTGAATGGA |
| VEGFA binding site 9 antisense: | ATGAAGGGGAAGCTCGACCC |
